# Data on hamster LD50 from Leptospira and its impact on Title 9, Codified Federal Regulations Sections 113.102–113.103 test validity

**DOI:** 10.1016/j.dib.2018.10.031

**Published:** 2018-10-16

**Authors:** Angela Walker, Renee Olsen, Mindy Toth, Larry Ludemann

**Affiliations:** Center for Veterinary Biologics, Animal and Plant Health Inspection Service, United States Department of Agriculture, P.O Box 844, Ames, IA 50010, USA

## Abstract

These data and analyses support the research article “Re-evaluating the LD50 requirements in the codified potency testing of veterinary vaccines containing *Leptospira* serogroups Icterohaemorrhagiae and Canicola in the United States” (Walker et al., 2018). Validity and disposition requirements submitted to the Center for Veterinary Biologics (CVB) are provided for serials (numbered lots) of commercial product potency tested for serogroups Canicola and Icterohaemorrhagiae in support of the Virus-Serum-Toxin Act (VSTA). Time course data for hamster loss after challenge with various concentrations of *Leptospira* during codified potency testing are also presented. The dose of *Leptospira* lethal to 50% of hamsters (LD50) was calculated by the Dragstedt-Behrens method for the *in vivo* data collected, and the equation is described here.

**Specifications table**

**Table:** 

Subject area	*Biology*
More specific subject area	*Regulatory Testing of Veterinary Vaccines*
Type of data	*Table and Figures, Raw and Analyzed*
How data was acquired	*APHIS Form 2008 Submissions*
	*9 CFR 113.102 – 113.103 Regulatory Potency Tests*
Data format	*Raw and analyzed*
Experimental factors	*in vivo Assays: Hamsters were either immunized with leptospiral bacterins or left as unvaccinated controls and, after a wait period, inoculated with Leptospira*
	*Retrospective Analysis: Data were analyzed for regulatory validity requirements including LD50 and valid challenge controls*
Experimental features	*in vivo Assays: Survivors were counted through twice daily observations.*
	*Retrospective Analysis: All confidential business information was redacted*
Data source location	*Ames, Iowa, USA or Animal Plant and Health Inspection Archives, USA*
Data accessibility	*Data are present in this article.*
Related research article	[1] Angela Walker, Renee Olsen, Mindy Toth, Geetha Srinivas. Re-evaluating the LD50 requirements in the codified potency testing of veterinary vaccines containing *Leptospira (L.)* serogroup Icterohaemorrhagiae and *L*. serogoup Canicola in the United States. Biologicals. 2018 (In Press). https://www.sciencedirect.com/science/article/pii/S1045105618302665?via%3Dihub.

**Value of the data**

• The retrospective analysis data can be used by policy makers, managers, and all related stakeholders, companies, agencies, and institutes in conjunction with regulatory testing data in other countries to assist global harmonization decisions for veterinary vaccine release.

• Data associated with regulatory leptospiral vaccine testing is accessible to basic researchers.
• Survival time course data for hamsters treated with various immunizations and challenge inoculums are provided. These may be used by other research groups in understanding the LD50 and disease progression variables for leptospirosis.

## 1 Data

**Fig. 1:**
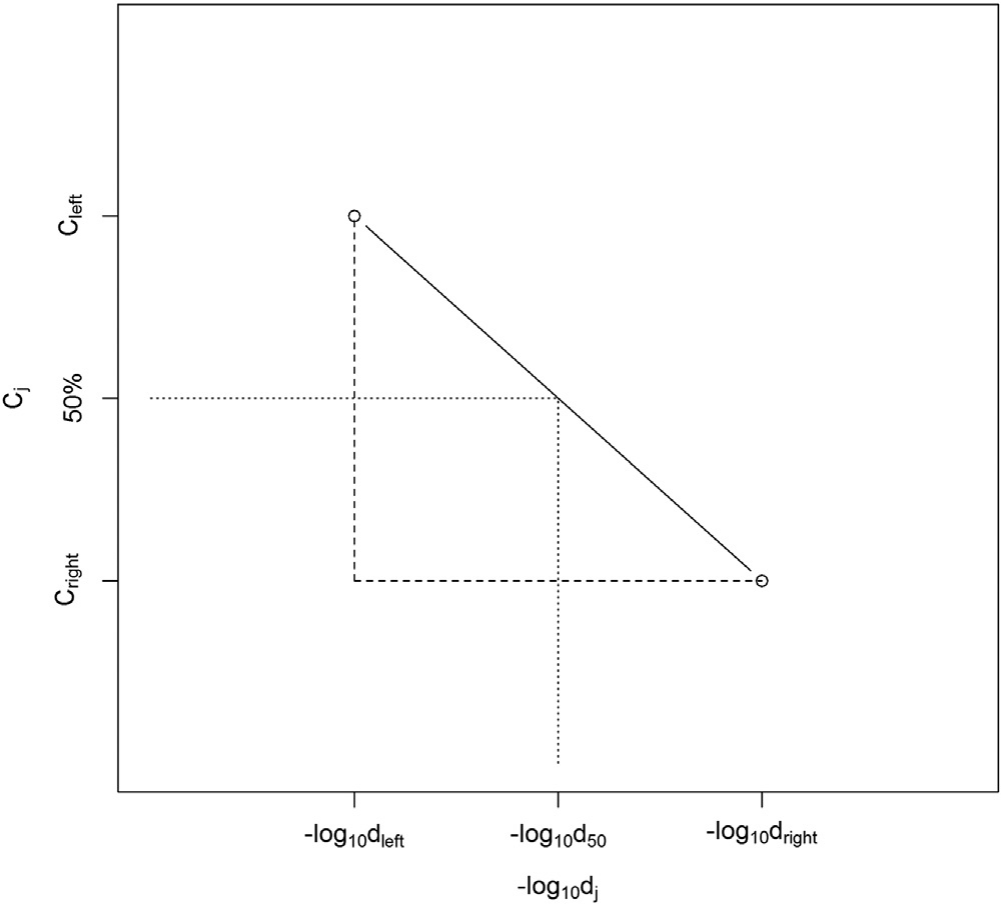
Illustration of linear interpolation to obtain LD50. The solid line connects the data points bracketing the 50% response, which form a right triangle outlined using dashed lines. The dotted lines indicate the 50% response and corresponding dilution.

**Fig. 2:**
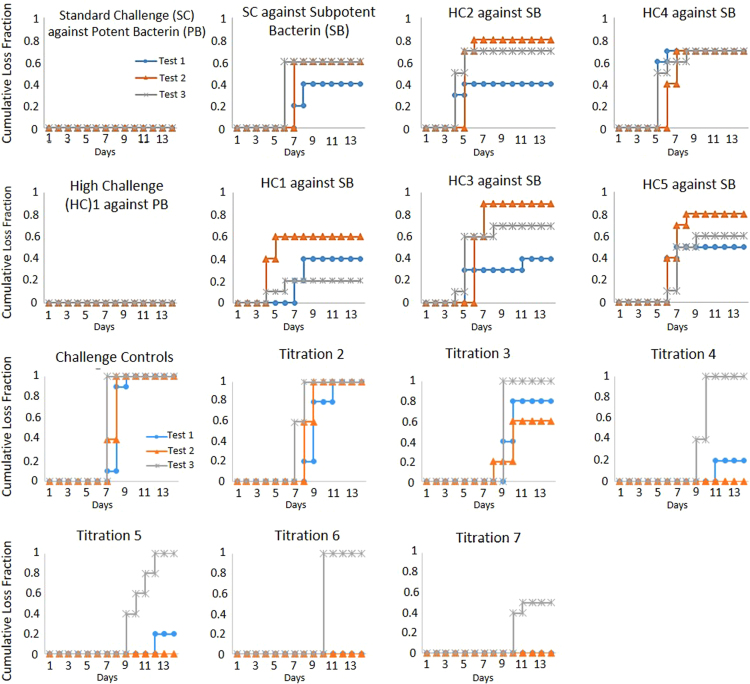
*Kaplan–Meier Loss Curves for L. serogroup Canicola.* The cumulative fraction of hamsters that succumbed to leptospirosis are shown over the 14 day observational period for each treatment group. The hamsters vaccinated with either potent bacterin (PB) or subpotent bacterin (SB) are illustrated in the first two rows while the unvaccinated controls given serial titrations of challenge are in the bottom two rows.

**Fig. 3:**
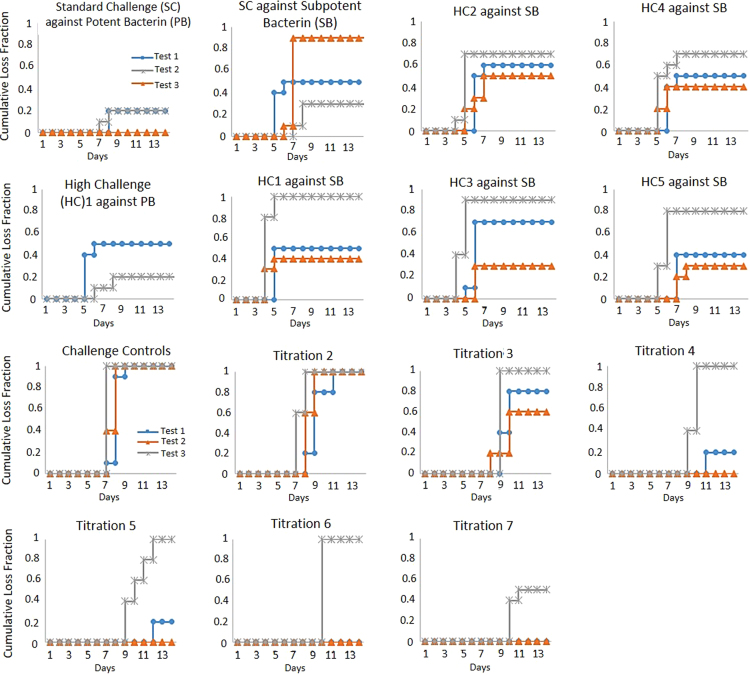
*Kaplan–Meier Loss Curves for L. serogroup Icterohaemorrhagiae.* The cumulative fraction of hamsters that succumbed to leptospirosis are shown over the 14 day observational period for each treatment group. The hamsters vaccinated with either potent bacterin (PB) or subpotent bacterin (SB) are illustrated in the first two rows while the unvaccinated controls given serial titrations of challenge are in the bottom two rows.

The codified leptospiral potency assays require ten hamsters (Harlan Sprague Dawley) to be vaccinated, and approximately two weeks later, the vaccinates and ten unvaccinated controls receive the same leptospiral challenge. Additional unvaccinated controls receive serial titrations of the leptospiral challenge to calculate the LD50. For a valid test, eight of ten controls receiving the same challenge inoculum as the vaccinates must succumb to disease and serial titration of the challenge must yield an LD50 of 10–10,000. The data presented extends the reproducibility of the recently published work exploring the validity requirements of codified potency testing of leptospiral vaccines [1]. Table 1 provides raw retrospective validity and disposition data on Title 9, Code of Federal Regulations (9 CFR) 113.102 – 113.103 potency testing for serial release. Fig. 1 depicts the formula for LD50 determination. Tables 2 and 3 contain the survival data for hamsters vaccinated with both potent and subpotent bacterins after receiving a range of leptospiral concentrations. It also includes the back-titrations used to determine LD50 for each test. Fig. 2, Fig. 3 are Kaplan–Meier curves of mortality over time for the *in vivo* experiments in Tables 2 and 3.

## 2 Experimental design, materials, and methods

### 2.1 Retrospective data

Serial release testing between July 2011 and April 2015 of vaccines containing *Leptospira* serogroups Canicola and Icterohaemorrhagiae fractions were submitted to the CVB according to 9 CFR 116. Each entry represents one stage of a 9 CFR potency test, and entries were removed if (1) the incorrect number of animals were used in the challenge group, (2) the serial was destroyed causing the submitted data to potentially be incomplete, (3) animals escaped housing during testing, (4) animals were ill from other causes at the time of testing, or (5) the LD50 was not reported. No confidential business information including veterinary biologics manufacturer׳s identification, testing location, or testing dates was listed.

### 2.2 Vaccination-challenge assays and LD50 determination

Supplemental Assay Methods (SAMs) 609 and 610 describe the hamster potency tests for *Leptospira interrogans* serogroup Canicola and *Leptospira interrogans* serogroup Icterohaemorrhagiae bacterins [2], [3]. Specific immunization procedures for this data are described elsewhere [1]. SAMs 609 and 610 require LD50 estimation via Reed–Muench, Dragstedt–Behrens, or Spearman–Karber. For this work, the Dragstedt–Behrens method described here was used [4], [5]. This is not a general explanation of the Dragstedt–Behrens method, which can accommodate more general dilution sequences, including unequally spaced dilutions. Rather the following explanation is specific to the accompanying data and the work reported in this paper, which uses ten-fold serial dilutions. For this calculation:

Let *A*_*j*_ = the cumulative deaths for dilution *j*, from most dilute to least dilute.
Let *B*_*j*_ = the cumulative survivors for dilution *j*, from least dilute to most dilute.
Let the cumulative percent dead at dilution *j* be $C_{j}=\frac{A_{j}}{A_{j}+B_{j}}\times 100\%$
Define *C*_*left*_ as the lowest *C*_*j*_ > 50%, and define *C*_*right*_ as the highest *C*_*j*_ less than 50%.
Let *d*_*left*_ and *d*_*right*_ be the corresponding dilutions, which are the dilutions that bracket the 50% cumulative response, whose unknown dilution we call *d*_50_.

$$\mathrm{Finally}\;\mathrm{define}\;\beta \;=\;\frac{C_{\mathrm{left}}\;-\;50}{C_{\mathrm{left}}\;-\;C_{\mathrm{right}}}$$

Fig. 1 describes the geometry of the linear interpolation between the two bracketing dilutions. Using Fig. 1 and a geometrical relationship relating the lengths of the sides of similar right triangles, we find that $\frac{\left(-\log_{10}d_{50}\right)-(-\log_{10}d_{\mathrm{left}})}{(-\log_{10}d_{\mathrm{right}})-(-\log_{10}d_{\mathrm{left}})}=\frac{C_{\mathrm{left}}-50}{C_{\mathrm{left}}-C_{\mathrm{right}}}=\beta$. Since *d*_*right*_ and *d*_*left*_ are adjacent dilutions, their difference is one on the log scale, so the denominator of the left hand side is unity and the equation is simplified. Solving for $-\log_{10}d_{50}$ we thus obtain $-\log_{10}d_{50}=-\log_{10}d_{\mathrm{left}}+\beta$. Consequently, the LD50 is simply *d*_50_ where ${\mathrm{LD}50=d}_{50}=\frac{d_{\mathrm{left}}}{10^{\beta }}$.

It is sometimes expressed in reciprocal form, 1:LD50, the inverse of the above expression. To calculate number of LD50 received per animal, divide the inverse of the LD50 by the reciprocal of the challenge dilution. An example calculation is helpful. Consider the following challenge titration data from the serogroup Canicola test examining a marginally potent vaccine [1]:

**Table:** 

Dilution	#Dead/#Challenged	#Survived/#Challenged	#Cumulative dead ↑ (*A*_*j*_)	#Cumulative survived ↓ (*B*_*j*_)	Cumulative Dead/Cum. Total	Cumulative % Dead (*C*_*j*_)
10^−7^	10/10	0/10	21	0	21/21	100
10^−8^	4/5	1/5	11	1	11/12	91.7
10^−9^	4/5	1/5	7	2	7/9	77.8
10^−10^	3/5	2/5	3	4	3/7	42.9
10^−11^	0/5	5/5	0	9	0/9	0
10^−12^	0/5	5/5	0	14	0/14	0
10^−13^	0/5	5/5	0	19	0/19	0

Here *C*_*left*_ = 77.8, *d*_*left*_ = 10^−9^, *C*_*right*_ = 42.9, and *d*_*right*_ = 10^−10^. Accordingly, β = 0.795, and 1:LD50 = 6.24 × 10^8^. The challenge dilution is 10^−7^ so the LD50 per animal is 624.

Since the challenge dilution and the *d*_*left*_ are always the same during regulatory leptospiral potency testing, the exact dilutions cancel each other when calculating LD50 per animal. A series of tenfold serial dilutions from the standard challenge during titrations will always allow calculation of LD50 per animal regardless of the specific challenge dilution from the liver homogenate used.

## Appendix A Supplementary material

**Table 1.** Data on Title 9, Code of Federal Regulations (9 CFR) 113.102 – 113.103 potency testing for serial release. Challenge control and LD50 validity requirements as well as disposition for serials released between July 2011 and April 2015 are listed.

.

**Table 2. Survival Table for L. serogroup Canicola.** The raw data for losses per day, calculated cumulative loss and fractional loss for each treatment group over the 14 day observational period are presented for serogroup Canicola.

.

**Table 3. Survival Table for L. serogroup Icterohaemorrhagiae.** The raw data for losses per day, calculated cumulative loss and fractional loss for each treatment group over the 14 day observational period are presented for Icterohaemorrhagiae.
