# Supplementary material for: Data on hamster LD50 from Leptospira and its impact on Title 9, Codified Federal Regulations Sections 113.102–113.103 test validity
Source: Data Brief. 2018 Oct 16;21:1352–7. doi: 10.1016/j.dib.2018.10.031 (PMC6230966; doi:10.1016/j.dib.2018.10.031)
Supplement: Supplementary file 1 — Supplementary material [file mmc1.docx]

**Conflict of Interest and Author Agreement Statement**

We confirm that we have given due consideration to the protection of intellectual property associated with this work and that there are no impediments to publication, including the timing of publication, with respect to intellectual property. In so doing we confirm that we have followed the regulations of our institutions concerning intellectual property. No external funding was used for this project.

We further confirm that any aspect of the work covered in this manuscript that has involved either experimental animals has been conducted with the ethical approval of all relevant bodies and that such approvals are acknowledged within the manuscript.

We understand that the Corresponding Author, Angela Walker, is the sole contact for the Editorial process (including Editorial Manager and direct communications with the office). She is responsible for communicating with the other authors about progress, submissions of revisions and final approval of proofs. We confirm that we have provided a current, correct email address which is accessible by the Corresponding Author and which has been configured to accept email from Angela.M.Walker@aphis.usda.gov
