# Supplementary material for: Data on hamster LD50 from Leptospira and its impact on Title 9, Codified Federal Regulations Sections 113.102–113.103 test validity
Source: Data Brief. 2018 Oct 16;21:1352–7. doi: 10.1016/j.dib.2018.10.031 (PMC6230966; doi:10.1016/j.dib.2018.10.031)
Supplement: Supplementary file 2 — Table 1. Data on Title 9, Code of Federal Regulations (9 CFR) 113.102 – 113.103 potency testing for serial release. Challenge control and LD50 validity requirements as well as disposition for serials released between July 2011 and April 2015 are listed. [file mmc2.docx]

| **Canicola** | | | |  | **Icterohaemorrhagiae** | | | |
| --- | --- | --- | --- | --- | --- | --- | --- | --- |
| **No. Challenged** | **Challenge Survived** | **LD50** | **Disposition** |  | **No. Challenged** | **Challenge Survived** | **LD50** | **Disposition** |
| 10 | 0 | 178 | SATISFACTORY |  | 10 | 0 | 699 | SATISFACTORY |
| 10 | 0 | 316 | SATISFACTORY |  | 10 | 0 | 2089 | SATISFACTORY |
| 10 | 0 | 1738 | SATISFACTORY |  | 10 | 1 | 794 | SATISFACTORY |
| 10 | 0 | 3162 | SATISFACTORY |  | 10 | 0 | 794 | SATISFACTORY |
| 10 | 0 | 10000 | SATISFACTORY |  | 10 | 0 | 3162 | SATISFACTORY |
| 10 | 0 | 491 | SATISFACTORY |  | 10 | 1 | 1000 | SATISFACTORY |
| 10 | 0 | 464 | SATISFACTORY |  | 10 | 1 | 59 | SATISFACTORY |
| 10 | 0 | 3162 | SATISFACTORY |  | 10 | 0 | 50 | SATISFACTORY |
| 10 | 0 | 12589 | NO TEST |  | 10 | 0 | 1778 | SATISFACTORY |
| 10 | 0 | 316 | SATISFACTORY |  | 10 | 1 | 1000 | SATISFACTORY |
| 10 | 0 | 422 | SATISFACTORY |  | 10 | 0 | 1778 | SATISFACTORY |
| 10 | 0 | 240 | SATISFACTORY |  | 10 | 0 | 1000 | SATISFACTORY |
| 10 | 1 | 18 | SATISFACTORY |  | 10 | 0 | 3162 | SATISFACTORY |
| 10 | 1 | 42 | SATISFACTORY |  | 10 | 0 | 3511 | SATISFACTORY |
| 10 | 0 | 3162 | SATISFACTORY |  | 10 | 0 | 422 | SATISFACTORY |
| 10 | 0 | 562 | SATISFACTORY |  | 10 | 1 | 351 | SATISFACTORY |
| 10 | 0 | 4266 | SATISFACTORY |  | 10 | 0 | 4217 | SATISFACTORY |
| 10 | 0 | 3162 | SATISFACTORY |  | 10 | 0 | 2276 | SATISFACTORY |
| 10 | 0 | 398 | SATISFACTORY |  | 10 | 0 | 794 | SATISFACTORY |
| 10 | 0 | 2154 | SATISFACTORY |  | 10 | 0 | 316 | SATISFACTORY |
| 10 | 1 | 2154 | SATISFACTORY |  | 10 | 0 | 562 | SATISFACTORY |
| 10 | 0 | 316 | SATISFACTORY |  | 10 | 0 | 316 | SATISFACTORY |
| 10 | 0 | 1000 | SATISFACTORY |  | 10 | 1 | 228 | SATISFACTORY |
| 10 | 0 | 215 | SATISFACTORY |  | 10 | 0 | 3162 | SATISFACTORY |
| 10 | 0 | 3162 | SATISFACTORY |  | 10 | 0 | 56 | SATISFACTORY |
| 10 | 1 | 2154 | SATISFACTORY |  | 10 | 0 | 676 | SATISFACTORY |
| 10 | 0 | 562 | SATISFACTORY |  | 10 | 0 | 2154 | SATISFACTORY |
| 10 | 1 | 2154 | SATISFACTORY |  | 10 | 0 | 68 | SATISFACTORY |
| 10 | 1 | 422 | SATISFACTORY |  | 10 | 0 | 3162 | SATISFACTORY |
| 10 | 1 | 18 | SATISFACTORY |  | 10 | 0 | 1468 | SATISFACTORY |
| 10 | 0 | 3162 | SATISFACTORY |  | 10 | 0 | 215 | SATISFACTORY |
| 10 | 1 | 234 | SATISFACTORY |  | 10 | 0 | 562 | SATISFACTORY |
| 10 | 0 | 1778 | SATISFACTORY |  | 10 | 0 | 1794 | SATISFACTORY |
| 10 | 0 | 481 | SATISFACTORY |  | 10 | 0 | 3020 | SATISFACTORY |
| 10 | 0 | 240 | SATISFACTORY |  | 10 | 0 | 2344 | SATISFACTORY |
| 10 | 0 | 562 | SATISFACTORY |  | 10 | 0 | 1479 | SATISFACTORY |
| 10 | 1 | 178 | SATISFACTORY |  | 10 | 0 | 1000 | SATISFACTORY |
| 10 | 0 | 3162 | SATISFACTORY |  | 10 | 1 | 1468 | SATISFACTORY |
| 10 | 1 | 1445 | SATISFACTORY |  | 10 | 0 | 676 | SATISFACTORY |
| 10 | 0 | 6043 | SATISFACTORY |  | 10 | 1 | 59 | SATISFACTORY |
| 10 | 0 | 3162 | SATISFACTORY |  | 10 | 0 | 178 | SATISFACTORY |
| 10 | 0 | 178 | SATISFACTORY |  | 10 | 0 | 1334 | SATISFACTORY |
| 10 | 0 | 32 | SATISFACTORY |  | 10 | 2 | 40 | SATISFACTORY |
| 10 | 0 | 1778 | SATISFACTORY |  | 10 | 0 | 464 | SATISFACTORY |
| 10 | 0 | 398 | SATISFACTORY |  | 10 | 0 | 1778 | SATISFACTORY |
| 10 | 0 | 1738 | SATISFACTORY |  | 10 | 2 | 228 | SATISFACTORY |
| 10 | 0 | 692 | SATISFACTORY |  | 10 | 0 | 1608 | SATISFACTORY |
| 10 | 0 | 3162 | SATISFACTORY |  | 10 | 0 | 3162 | SATISFACTORY |
| 10 | 0 | 562 | SATISFACTORY |  | 10 | 1 | 1000 | SATISFACTORY |
| 10 | 1 | 1000 | SATISFACTORY |  | 10 | 1 | 351 | SATISFACTORY |
| 10 | 1 | 692 | SATISFACTORY |  | 10 | 0 | 558 | SATISFACTORY |
| 10 | 0 | 10000 | SATISFACTORY |  | 10 | 0 | 40 | SATISFACTORY |
| 10 | 1 | 151 | SATISFACTORY |  | 10 | 0 | 1778 | SATISFACTORY |
| 10 | 0 | 200 | SATISFACTORY |  | 10 | 0 | 209 | SATISFACTORY |
| 10 | 0 | 7943 | SATISFACTORY |  | 10 | 0 | 1000 | SATISFACTORY |
| 10 | 0 | 478 | SATISFACTORY |  | 10 | 0 | 1778 | SATISFACTORY |
| 10 | 0 | 422 | SATISFACTORY |  | 10 | 0 | 1778 | NO TEST |
| 10 | 0 | 126 | SATISFACTORY |  | 10 | 0 | 1778 | SATISFACTORY |
| 10 | 0 | 3162 | SATISFACTORY |  | 10 | 1 | 228 | NO TEST |
| 10 | 1 | 1778 | SATISFACTORY |  | 10 | 0 | 2154 | SATISFACTORY |
| 10 | 0 | 562 | SATISFACTORY |  | 10 | 0 | 550 | SATISFACTORY |
| 10 | 0 | 1000 | SATISFACTORY |  | 10 | 0 | 316 | SATISFACTORY |
| 10 | 0 | 182 | SATISFACTORY |  | 10 | 0 | 347 | SATISFACTORY |
| 10 | 1 | 346 | SATISFACTORY |  | 10 | 0 | 1000 | SATISFACTORY |
| 10 | 0 | 692 | SATISFACTORY |  | 10 | 0 | 3162 | SATISFACTORY |
| 10 | 1 | 1778 | SATISFACTORY |  | 10 | 0 | 464 | SATISFACTORY |
| 10 | 0 | 1000 | SATISFACTORY |  | 10 | 0 | 1000 | SATISFACTORY |
| 10 | 0 | 1000 | SATISFACTORY |  | 10 | 0 | 1778 | SATISFACTORY |
| 10 | 0 | 1778 | SATISFACTORY |  | 10 | 1 | 700 | SATISFACTORY |
| 10 | 0 | 237 | SATISFACTORY |  | 10 | 0 | 474 | SATISFACTORY |
| 10 | 0 | 2581 | SATISFACTORY |  | 10 | 0 | 492 | SATISFACTORY |
| 10 | 0 | 562 | SATISFACTORY |  | 10 | 0 | 2093 | SATISFACTORY |
| 10 | 0 | 316 | SATISFACTORY |  | 10 | 0 | 1468 | SATISFACTORY |
| 10 | 0 | 6813 | SATISFACTORY |  | 10 | 0 | 3162 | SATISFACTORY |
| 10 | 0 | 4786 | SATISFACTORY |  | 10 | 0 | 2371 | SATISFACTORY |
| 10 | 2 | 15 | SATISFACTORY |  | 10 | 0 | 3162 | SATISFACTORY |
| 10 | 0 | 178 | SATISFACTORY |  | 10 | 1 | 178 | SATISFACTORY |
| 10 | 0 | 178 | SATISFACTORY |  | 10 | 0 | 1000 | SATISFACTORY |
| 10 | 0 | 1000 | NO TEST |  | 10 | 0 | 588 | SATISFACTORY |
| 10 | 0 | 562 | SATISFACTORY |  | 10 | 0 | 1000 | SATISFACTORY |
| 10 | 0 | 316 | SATISFACTORY |  | 10 | 0 | 316 | SATISFACTORY |
| 10 | 0 | 316 | SATISFACTORY |  | 10 | 0 | 1585 | SATISFACTORY |
| 10 | 0 | 464 | SATISFACTORY |  | 10 | 0 | 3162 | SATISFACTORY |
| 10 | 0 | 46 | SATISFACTORY |  | 10 | 1 | 228 | SATISFACTORY |
| 10 | 1 | 42 | SATISFACTORY |  | 10 | 0 | 3511 | SATISFACTORY |
| 10 | 1 | 1000 | SATISFACTORY |  | 10 | 2 | 126 | SATISFACTORY |
| 10 | 0 | 3162 | SATISFACTORY |  | 10 | 1 | 1000 | SATISFACTORY |
| 10 | 0 | 3162 | SATISFACTORY |  | 10 | 0 | 316 | SATISFACTORY |
| 10 | 0 | 316 | SATISFACTORY |  | 10 | 0 | 1259 | SATISFACTORY |
| 10 | 1 | 159 | SATISFACTORY |  | 10 | 0 | 562 | SATISFACTORY |
| 10 | 0 | 178 | SATISFACTORY |  | 10 | 0 | 56 | SATISFACTORY |
| 10 | 0 | 1479 | SATISFACTORY |  | 10 | 3 | 599 | NO TEST |
| 10 | 0 | 681 | SATISFACTORY |  | 10 | 0 | 1778 | SATISFACTORY |
| 10 | 0 | 562 | SATISFACTORY |  | 10 | 0 | 681 | SATISFACTORY |
| 10 | 0 | 681 | SATISFACTORY |  | 10 | 1 | 794 | SATISFACTORY |
| 10 | 1 | 481 | SATISFACTORY |  | 10 | 0 | 1778 | SATISFACTORY |
| 10 | 0 | 562 | SATISFACTORY |  | 10 | 1 | 178 | SATISFACTORY |
| 10 | 0 | 562 | SATISFACTORY |  | 10 | 0 | 1995 | SATISFACTORY |
| 10 | 3 | 20 | NO TEST |  | 10 | 0 | 351 | SATISFACTORY |
| 10 | 0 | 100 | SATISFACTORY |  | 10 | 0 | 1778 | SATISFACTORY |
| 10 | 1 | 1318 | SATISFACTORY |  | 10 | 1 | 234 | SATISFACTORY |
| 10 | 1 | 351 | SATISFACTORY |  | 10 | 0 | 215 | SATISFACTORY |
| 10 | 0 | 398 | SATISFACTORY |  | 10 | 0 | 158 | SATISFACTORY |
| 10 | 0 | 215 | SATISFACTORY |  | 10 | 0 | 562 | SATISFACTORY |
| 10 | 1 | 351 | SATISFACTORY |  | 10 | 0 | 3162 | SATISFACTORY |
| 10 | 1 | 481 | SATISFACTORY |  | 10 | 0 | 1000 | SATISFACTORY |
| 10 | 0 | 32 | SATISFACTORY |  | 10 | 0 | 1778 | SATISFACTORY |
| 10 | 0 | 681 | SATISFACTORY |  | 10 | 0 | 2276 | SATISFACTORY |
| 10 | 0 | 681 | SATISFACTORY |  | 10 | 0 | 4217 | SATISFACTORY |
| 10 | 0 | 562 | SATISFACTORY |  | 10 | 1 | 501 | SATISFACTORY |
| 10 | 0 | 1778 | SATISFACTORY |  | 10 | 1 | 316 | SATISFACTORY |
| 10 | 0 | 681 | SATISFACTORY |  | 10 | 0 | 3162 | SATISFACTORY |
| 10 | 1 | 178 | SATISFACTORY |  | 10 | 0 | 1000 | SATISFACTORY |
| 10 | 1 | 481 | SATISFACTORY |  | 10 | 0 | 1778 | SATISFACTORY |
| 10 | 0 | 316 | SATISFACTORY |  | 10 | 0 | 464 | SATISFACTORY |
| 10 | 0 | 178 | SATISFACTORY |  | 10 | 1 | 215 | SATISFACTORY |
| 10 | 1 | 147 | SATISFACTORY |  | 10 | 1 | 1000 | SATISFACTORY |
| 10 | 0 | 681 | SATISFACTORY |  | 10 | 0 | 464 | SATISFACTORY |
| 10 | 0 | 1000 | SATISFACTORY |  | 10 | 0 | 1000 | SATISFACTORY |
| 10 | 0 | 215 | SATISFACTORY |  | 10 | 1 | 316 | SATISFACTORY |
| 10 | 1 | 1445 | SATISFACTORY |  | 10 | 0 | 422 | SATISFACTORY |
| 10 | 0 | 159 | SATISFACTORY |  | 10 | 1 | 178 | SATISFACTORY |
| 10 | 0 | 316 | SATISFACTORY |  | 10 | 0 | 1349 | SATISFACTORY |
| 10 | 1 | 4677 | SATISFACTORY |  | 10 | 1 | 178 | SATISFACTORY |
| 10 | 0 | 351 | SATISFACTORY |  | 10 | 0 | 588 | SATISFACTORY |
| 10 | 0 | 501 | SATISFACTORY |  | 10 | 0 | 3162 | SATISFACTORY |
| 10 | 2 | 15 | SATISFACTORY |  | 10 | 2 | 228 | SATISFACTORY |
| 10 | 0 | 215 | SATISFACTORY |  | 10 | 1 | 700 | SATISFACTORY |
| 10 | 0 | 46 | SATISFACTORY |  | 10 | 0 | 237 | SATISFACTORY |
| 10 | 0 | 3162 | SATISFACTORY |  | 10 | 1 | 231 | SATISFACTORY |
| 10 | 0 | 316 | SATISFACTORY |  | 10 | 0 | 50 | SATISFACTORY |
| 10 | 0 | 316 | SATISFACTORY |  | 10 | 0 | 1000 | SATISFACTORY |
| 10 | 2 | 76 | SATISFACTORY |  | 10 | 0 | 3162 | SATISFACTORY |
| 10 | 0 | 481 | SATISFACTORY |  | 10 | 0 | 316 | SATISFACTORY |
| 10 | 0 | 200 | SATISFACTORY |  | 10 | 1 | 2154 | SATISFACTORY |
| 10 | 0 | 562 | SATISFACTORY |  | 10 | 0 | 3162 | SATISFACTORY |
| 10 | 1 | 422 | SATISFACTORY |  | 10 | 0 | 3511 | SATISFACTORY |
| 10 | 1 | 351 | SATISFACTORY |  | 10 | 1 | 1000 | SATISFACTORY |
| 10 | 0 | 422 | SATISFACTORY |  | 10 | 0 | 351 | SATISFACTORY |
| 10 | 0 | 562 | SATISFACTORY |  | 10 | 0 | 1778 | SATISFACTORY |
| 10 | 0 | 1000 | SATISFACTORY |  | 10 | 0 | 1096 | SATISFACTORY |
| 10 | 1 | 100 | SATISFACTORY |  | 10 | 0 | 3162 | SATISFACTORY |
| 10 | 0 | 178 | SATISFACTORY |  | 10 | 0 | 351 | SATISFACTORY |
| 10 | 0 | 794 | SATISFACTORY |  | 10 | 0 | 3162 | SATISFACTORY |
| 10 | 0 | 3162 | SATISFACTORY |  | 10 | 0 | 562 | SATISFACTORY |
| 10 | 1 | 42 | SATISFACTORY |  | 10 | 0 | 1000 | SATISFACTORY |
| 10 | 0 | 681 | SATISFACTORY |  | 10 | 0 | 3020 | SATISFACTORY |
| 10 | 1 | 100 | SATISFACTORY |  | 10 | 0 | 55 | SATISFACTORY |
| 10 | 0 | 562 | SATISFACTORY |  | 10 | 0 | 1581 | SATISFACTORY |
| 10 | 0 | 1778 | SATISFACTORY |  | 10 | 0 | 1778 | SATISFACTORY |
| 10 | 0 | 3162 | SATISFACTORY |  | 10 | 0 | 464 | SATISFACTORY |
| 10 | 0 | 3162 | SATISFACTORY |  | 10 | 0 | 3511 | SATISFACTORY |
| 10 | 0 | 464 | SATISFACTORY |  | 10 | 0 | 1778 | SATISFACTORY |
| 10 | 1 | 234 | SATISFACTORY |  | 10 | 0 | 702 | SATISFACTORY |
| 10 | 1 | 215 | SATISFACTORY |  | 10 | 0 | 955 | SATISFACTORY |
| 10 | 0 | 1778 | SATISFACTORY |  | 10 | 0 | >10000 | NO TEST |
| 10 | 0 | 481 | Inconclusive |  | 10 | 1 | 234 | SATISFACTORY |
| 10 | 1 | 351 | SATISFACTORY |  | 10 | 0 | 2512 | SATISFACTORY |
| 10 | 0 | 316 | SATISFACTORY |  | 10 | 0 | 422 | SATISFACTORY |
| 10 | 0 | 3162 | SATISFACTORY |  | 10 | 0 | 3162 | SATISFACTORY |
| 10 | 0 | 1000 | SATISFACTORY |  | 10 | 1 | 2154 | SATISFACTORY |
| 10 | 1 | 147 | SATISFACTORY |  | 10 | 0 | 1000 | SATISFACTORY |
| 10 | 0 | 316 | SATISFACTORY |  | 10 | 0 | 681 | SATISFACTORY |
| 10 | 1 | 178 | SATISFACTORY |  | 10 | 0 | 562 | SATISFACTORY |
| 10 | 2 | 15 | SATISFACTORY |  | 10 | 0 | 316 | SATISFACTORY |
| 10 | 1 | 351 | SATISFACTORY |  | 10 | 0 | 1000 | SATISFACTORY |
| 10 | 1 | 481 | SATISFACTORY |  | 10 | 0 | 1778 | SATISFACTORY |
| 10 | 0 | 46 | SATISFACTORY |  | 10 | 1 | 1000 | SATISFACTORY |
| 10 | 1 | 100 | SATISFACTORY |  | 10 | 0 | 215 | SATISFACTORY |
| 10 | 0 | 3162 | SATISFACTORY |  | 10 | 1 | 178 | SATISFACTORY |
| 10 | 0 | 562 | SATISFACTORY |  | 10 | 1 | 422 | SATISFACTORY |
| 10 | 2 | 76 | SATISFACTORY |  | 10 | 1 | 481 | SATISFACTORY |
| 10 | 0 | 794 | SATISFACTORY |  | 10 | 1 | 215 | SATISFACTORY |
| 10 | 0 | 100 | SATISFACTORY |  | 10 | 0 | 1778 | SATISFACTORY |
| 10 | 1 | 422 | SATISFACTORY |  | 10 | 0 | 562 | SATISFACTORY |
| 10 | 0 | 178 | SATISFACTORY |  | 10 | 0 | 1778 | SATISFACTORY |
| 10 | 0 | 427 | SATISFACTORY |  | 10 | 0 | 215 | SATISFACTORY |
| 10 | 0 | 178 | SATISFACTORY |  | 10 | 0 | 237 | SATISFACTORY |
| 10 | 0 | 316 | SATISFACTORY |  | 10 | 1 | 316 | SATISFACTORY |
| 10 | 0 | 178 | SATISFACTORY |  | 10 | 0 | 3162 | SATISFACTORY |
| 10 | 0 | 2154 | SATISFACTORY |  | 10 | 0 | 178 | SATISFACTORY |
| 10 | 2 | 15 | SATISFACTORY |  | 10 | 0 | 1202 | SATISFACTORY |
| 10 | 1 | 100 | SATISFACTORY |  | 10 | 0 | 6310 | SATISFACTORY |
| 10 | 0 | 676 | SATISFACTORY |  | 10 | 1 | 1000 | SATISFACTORY |
| 10 | 0 | 676 | SATISFACTORY |  | 10 | 0 | 1778 | SATISFACTORY |
| 10 | 0 | 316 | SATISFACTORY |  | 10 | 1 | 1000 | SATISFACTORY |
| 10 | 0 | 3495 | SATISFACTORY |  | 10 | 0 | 464 | SATISFACTORY |
| 10 | 0 | 481 | SATISFACTORY |  | 10 | 0 | 3162 | SATISFACTORY |
| 10 | 0 | 624 | SATISFACTORY |  | 10 | 0 | 1778 | SATISFACTORY |
| 10 | 1 | 4908 | SATISFACTORY |  | 10 | 0 | 351 | SATISFACTORY |
| 10 | 0 | 676 | SATISFACTORY |  | 10 | 0 | 3162 | SATISFACTORY |
| 10 | 0 | 681 | SATISFACTORY |  | 10 | 0 | 562 | SATISFACTORY |
| 10 | 0 | 562 | SATISFACTORY |  | 10 | 0 | 562 | SATISFACTORY |
| 10 | 1 | 346 | SATISFACTORY |  | 10 | 1 | 1995 | Inconclusive |
| 10 | 1 | 147 | SATISFACTORY |  | 10 | 0 | 1501 | SATISFACTORY |
| 10 | 1 | 1000 | SATISFACTORY |  | 10 | 0 | 3511 | SATISFACTORY |
| 10 | 1 | 351 | SATISFACTORY |  | 10 | 0 | 316 | SATISFACTORY |
| 10 | 0 | 1334 | SATISFACTORY |  | 10 | 1 | 316 | SATISFACTORY |
| 10 | 1 | 35 | SATISFACTORY |  | 10 | 0 | 174 | SATISFACTORY |
| 10 | 0 | 479 | SATISFACTORY |  | 10 | 0 | 2512 | SATISFACTORY |
| 10 | 0 | 316 | SATISFACTORY |  | 10 | 0 | 316 | SATISFACTORY |
| 10 | 1 | 42 | SATISFACTORY |  | 10 | 1 | 228 | SATISFACTORY |
| 10 | 0 | 316 | SATISFACTORY |  | 10 | 0 | 316 | SATISFACTORY |
| 10 | 1 | 2154 | SATISFACTORY |  | 10 | 0 | 562 | SATISFACTORY |
| 10 | 0 | 316 | SATISFACTORY |  | 10 | 0 | 562 | SATISFACTORY |
| 10 | 1 | 1468 | SATISFACTORY |  | 10 | 0 | 1778 | SATISFACTORY |
| 10 | 2 | 48 | SATISFACTORY |  | 10 | 0 | 316 | SATISFACTORY |
| 10 | 0 | 351 | SATISFACTORY |  | 10 | 1 | 257 | SATISFACTORY |
| 10 | 0 | 178 | SATISFACTORY |  | 10 | 1 | 234 | SATISFACTORY |
| 10 | 0 | 215 | SATISFACTORY |  | 10 | 0 | 3511 | SATISFACTORY |
| 10 | 0 | 178 | SATISFACTORY |  | 10 | 1 | 316 | SATISFACTORY |
| 10 | 1 | 215 | SATISFACTORY |  | 10 | 0 | 3162 | SATISFACTORY |
| 10 | 1 | 100 | SATISFACTORY |  | 10 | 1 | 2089 | SATISFACTORY |
| 10 | 0 | 2344 | SATISFACTORY |  | 10 | 1 | 13 | SATISFACTORY |
| 10 | 0 | 316 | SATISFACTORY |  | 10 | 0 | 1778 | SATISFACTORY |
| 10 | 0 | 1000 | SATISFACTORY |  | 10 | 0 | 1000 | SATISFACTORY |
| 10 | 0 | 32 | SATISFACTORY |  | 10 | 3 | 599 | NO TEST |
| 10 | 0 | 215 | SATISFACTORY |  | 10 | 0 | 464 | SATISFACTORY |
| 10 | 0 | 2154 | SATISFACTORY |  | 10 | 0 | 1334 | SATISFACTORY |
| 10 | 0 | 478 | SATISFACTORY |  | 10 | 1 | 1000 | SATISFACTORY |
| 10 | 0 | 240 | SATISFACTORY |  | 10 | 0 | 562 | SATISFACTORY |
| 10 | 1 | 100 | SATISFACTORY |  | 10 | 0 | 3162 | SATISFACTORY |
| 10 | 0 | 200 | SATISFACTORY |  | 10 | 0 | 351 | SATISFACTORY |
| 10 | 0 | 681 | SATISFACTORY |  | 10 | 1 | 59 | SATISFACTORY |
| 10 | 1 | 1778 | SATISFACTORY |  | 10 | 0 | 2371 | SATISFACTORY |
| 10 | 0 | 286 | SATISFACTORY |  | 10 | 1 | 1000 | SATISFACTORY |
| 10 | 0 | 794 | SATISFACTORY |  | 10 | 0 | 562 | SATISFACTORY |
| 10 | 1 | 18 | SATISFACTORY |  | 10 | 0 | 351 | SATISFACTORY |
| 10 | 1 | 215 | SATISFACTORY |  | 10 | 0 | 1000 | SATISFACTORY |
| 10 | 0 | 48 | SATISFACTORY |  | 10 | 0 | 562 | SATISFACTORY |
| 10 | 0 | 316 | SATISFACTORY |  | 10 | 0 | 3162 | SATISFACTORY |
| 10 | 0 | 215 | SATISFACTORY |  | 10 | 1 | 228 | SATISFACTORY |
| 10 | 1 | 147 | SATISFACTORY |  | 10 | 1 | 422 | SATISFACTORY |
| 10 | 0 | 48 | SATISFACTORY |  | 10 | 0 | 562 | SATISFACTORY |
| 10 | 1 | 422 | SATISFACTORY |  | 10 | 0 | 464 | SATISFACTORY |
| 10 | 0 | 79 | SATISFACTORY |  | 10 | 1 | 1000 | SATISFACTORY |
| 10 | 0 | 100 | SATISFACTORY |  | 10 | 0 | 3162 | SATISFACTORY |
| 10 | 0 | 1000 | SATISFACTORY |  | 10 | 0 | 316 | SATISFACTORY |
| 10 | 0 | 5623 | SATISFACTORY |  | 10 | 0 | 588 | SATISFACTORY |
| 10 | 0 | 692 | SATISFACTORY |  | 10 | 0 | 1000 | SATISFACTORY |
| 10 | 1 | 159 | SATISFACTORY |  | 10 | 0 | 3162 | SATISFACTORY |
| 10 | 1 | 692 | SATISFACTORY |  | 10 | 1 | 228 | SATISFACTORY |
| 10 | 0 | 286 | SATISFACTORY |  | 10 | 0 | 1000 | SATISFACTORY |
| 10 | 0 | 1000 | SATISFACTORY |  | 10 | 0 | 3162 | SATISFACTORY |
| 10 | 0 | 2154 | SATISFACTORY |  | 10 | 0 | 237 | SATISFACTORY |
| 10 | 1 | 178 | SATISFACTORY |  | 10 | 0 | 3162 | SATISFACTORY |
| 10 | 0 | 316 | SATISFACTORY |  | 10 | 0 | 68 | SATISFACTORY |
| 10 | 0 | 316 | SATISFACTORY |  | 10 | 0 | 562 | SATISFACTORY |
| 10 | 0 | 316 | SATISFACTORY |  | 10 | 0 | 316 | SATISFACTORY |
| 10 | 0 | 316 | SATISFACTORY |  | 10 | 0 | 351 | SATISFACTORY |
| 10 | 0 | 562 | SATISFACTORY |  | 10 | 0 | 1445 | SATISFACTORY |
| 10 | 0 | 2581 | SATISFACTORY |  | 10 | 0 | 1778 | SATISFACTORY |
| 10 | 0 | 3162 | SATISFACTORY |  | 10 | 0 | 3162 | SATISFACTORY |
| 10 | 0 | 562 | SATISFACTORY |  | 10 | 0 | 3162 | SATISFACTORY |
| 10 | 0 | 316 | SATISFACTORY |  | 10 | 0 | 316 | SATISFACTORY |
| 10 | 0 | 18 | SATISFACTORY |  | 10 | 1 | 422 | SATISFACTORY |
| 10 | 0 | 214 | SATISFACTORY |  | 10 | 0 | 1000 | SATISFACTORY |
| 10 | 0 | 1000 | SATISFACTORY |  | 10 | 1 | 1000 | SATISFACTORY |
| 10 | 0 | 316 | SATISFACTORY |  | 10 | 0 | 237 | SATISFACTORY |
| 10 | 0 | 692 | SATISFACTORY |  | 10 | 0 | 562 | SATISFACTORY |
| 10 | 0 | 428 | SATISFACTORY |  | 10 | 1 | 178 | SATISFACTORY |
| 10 | 0 | 427 | SATISFACTORY |  | 10 | 0 | 316 | SATISFACTORY |
| 10 | 0 | 179 | SATISFACTORY |  | 10 | 0 | 316 | SATISFACTORY |
| 10 | 0 | 3162 | SATISFACTORY |  | 10 | 0 | 3162 | Inconclusive |
| 10 | 0 | 562 | SATISFACTORY |  | 10 | 0 | 1778 | SATISFACTORY |
| 10 | 0 | 316 | SATISFACTORY |  | 10 | 1 | 316 | SATISFACTORY |
| 10 | 0 | 481 | SATISFACTORY |  | 10 | 0 | 562 | SATISFACTORY |
| 10 | 0 | 3162 | SATISFACTORY |  | 10 | 1 | 422 | SATISFACTORY |
| 10 | 1 | 3162 | SATISFACTORY |  | 10 | 0 | 316 | SATISFACTORY |
| 10 | 1 | 351 | SATISFACTORY |  | 10 | 0 | 3162 | SATISFACTORY |
| 10 | 0 | 562 | SATISFACTORY |  | 10 | 0 | 178 | SATISFACTORY |
| 10 | 0 | 3162 | SATISFACTORY |  | 10 | 0 | 178 | SATISFACTORY |
| 10 | 0 | 234 | SATISFACTORY |  | 10 | 1 | 179 | SATISFACTORY |
| 10 | 1 | 692 | SATISFACTORY |  | 10 | 0 | 178 | SATISFACTORY |
| 10 | 0 | 428 | SATISFACTORY |  | 10 | 0 | 1000 | SATISFACTORY |
| 10 | 2 | 2081 | SATISFACTORY |  | 10 | 0 | 1778 | SATISFACTORY |
| 10 | 0 | 562 | SATISFACTORY |  | 10 | 0 | 316 | SATISFACTORY |
| 10 | 0 | 1000 | SATISFACTORY |  | 10 | 0 | 1000 | SATISFACTORY |
| 10 | 0 | 1000 | SATISFACTORY |  | 10 | 0 | 3162 | SATISFACTORY |
| 10 | 0 | 316 | SATISFACTORY |  | 10 | 0 | 316 | SATISFACTORY |
| 10 | 2 | 316 | SATISFACTORY |  | 10 | 1 | 228 | SATISFACTORY |
| 10 | 0 | 5012 | SATISFACTORY |  | 10 | 0 | 351 | SATISFACTORY |
| 10 | 0 | 178 | SATISFACTORY |  | 10 | 0 | 562 | SATISFACTORY |
| 10 | 0 | 389 | SATISFACTORY |  | 10 | 0 | 4217 | SATISFACTORY |
| 10 | 0 | 464 | SATISFACTORY |  | 10 | 0 | 1585 | SATISFACTORY |
| 10 | 0 | 3162 | SATISFACTORY |  | 10 | 0 | 2371 | SATISFACTORY |
| 10 | 0 | 12589 | NO TEST |  | 10 | 0 | 1000 | SATISFACTORY |
| 10 | 0 | 56 | SATISFACTORY |  | 10 | 1 | 257 | SATISFACTORY |
| 10 | 2 | 23 | SATISFACTORY |  | 10 | 0 | 316 | SATISFACTORY |
| 10 | 0 | 10 | SATISFACTORY |  | 10 | 0 | 2512 | SATISFACTORY |
| 10 | 0 | 4786 | SATISFACTORY |  | 10 | 0 | 237 | SATISFACTORY |
| 10 | 1 | 481 | SATISFACTORY |  | 10 | 1 | 1000 | SATISFACTORY |
| 10 | 0 | 228 | SATISFACTORY |  | 10 | 0 | 562 | SATISFACTORY |
| 10 | 0 | 100 | SATISFACTORY |  | 10 | 1 | 1000 | SATISFACTORY |
| 10 | 0 | 10000 | SATISFACTORY |  | 10 | 0 | 562 | SATISFACTORY |
| 10 | 0 | 3162 | SATISFACTORY |  | 10 | 0 | 3162 | SATISFACTORY |
| 10 | 0 | 351 | SATISFACTORY |  | 10 | 0 | 3162 | SATISFACTORY |
| 10 | 0 | 316 | SATISFACTORY |  | 10 | 0 | 3162 | SATISFACTORY |
| 10 | 1 | 100 | SATISFACTORY |  | 10 | 0 | 699 | SATISFACTORY |
| 10 | 0 | 200 | SATISFACTORY |  | 10 | 0 | 562 | SATISFACTORY |
| 10 | 0 | 1000 | NO TEST |  | 10 | 0 | 2371 | SATISFACTORY |
| 10 | 0 | 351 | SATISFACTORY |  | 10 | 0 | 4217 | SATISFACTORY |
| 10 | 2 | 316 | SATISFACTORY |  | 10 | 0 | 215 | SATISFACTORY |
| 10 | 1 | 681 | SATISFACTORY |  | 10 | 0 | 3162 | SATISFACTORY |
| 10 | 0 | 32 | SATISFACTORY |  | 10 | 0 | 681 | SATISFACTORY |
| 10 | 0 | 2081 | SATISFACTORY |  | 10 | 0 | 3162 | SATISFACTORY |
| 10 | 0 | 1000 | SATISFACTORY |  | 10 | 0 | 1468 | SATISFACTORY |
| 10 | 1 | 42 | SATISFACTORY |  | 10 | 1 | 1000 | SATISFACTORY |
| 10 | 0 | 316 | SATISFACTORY |  | 10 | 0 | 464 | SATISFACTORY |
| 10 | 0 | 316 | SATISFACTORY |  | 10 | 0 | 1778 | SATISFACTORY |
| 10 | 0 | 18 | SATISFACTORY |  | 10 | 0 | 178 | SATISFACTORY |
| 10 | 1 | 55 | SATISFACTORY |  | 10 | 0 | 3511 | SATISFACTORY |
| 10 | 0 | 3162 | SATISFACTORY |  | 10 | 0 | 1000 | SATISFACTORY |
| 10 | 0 | 427 | SATISFACTORY |  | 10 | 2 | 418 | SATISFACTORY |
| 10 | 0 | 562 | SATISFACTORY |  | 10 | 2 | 52 | SATISFACTORY |
| 10 | 0 | 478 | SATISFACTORY |  | 10 | 0 | 398 | SATISFACTORY |
| 10 | 1 | 4908 | SATISFACTORY |  | 10 | 0 | 1778 | SATISFACTORY |
| 10 | 0 | 3162 | SATISFACTORY |  | 10 | 0 | 3511 | SATISFACTORY |
| 10 | 0 | 178 | SATISFACTORY |  | 10 | 0 | 1000 | SATISFACTORY |
| 10 | 1 | 18 | SATISFACTORY |  | 10 | 1 | 228 | SATISFACTORY |
| 10 | 1 | 1778 | SATISFACTORY |  | 10 | 0 | 316 | SATISFACTORY |
| 10 | 0 | 422 | SATISFACTORY |  | 10 | 0 | 3162 | SATISFACTORY |
| 10 | 0 | 351 | SATISFACTORY |  | 10 | 0 | 1000 | SATISFACTORY |
| 10 | 1 | 2540 | SATISFACTORY |  | 10 | 0 | 3162 | SATISFACTORY |
| 10 | 0 | 32 | SATISFACTORY |  | 10 | 1 | 631 | SATISFACTORY |
| 10 | 1 | 1468 | SATISFACTORY |  | 10 | 1 | 1000 | SATISFACTORY |
| 10 | 0 | 178 | SATISFACTORY |  | 10 | 0 | 316 | SATISFACTORY |
| 10 | 0 | 562 | SATISFACTORY |  | 10 | 0 | 562 | SATISFACTORY |
| 10 | 0 | 200 | SATISFACTORY |  | 10 | 0 | 351 | SATISFACTORY |
| 10 | 1 | 1000 | SATISFACTORY |  | 10 | 0 | 3162 | SATISFACTORY |
| 10 | 0 | 178 | SATISFACTORY |  | 10 | 0 | 1778 | SATISFACTORY |
| 10 | 0 | 316 | SATISFACTORY |  | 10 | 0 | 237 | SATISFACTORY |
| 10 | 0 | 350 | SATISFACTORY |  | 10 | 1 | 1000 | SATISFACTORY |
| 10 | 0 | 1468 | SATISFACTORY |  | 10 | 0 | 2371 | SATISFACTORY |
| 10 | 0 | 316 | SATISFACTORY |  | 10 | 0 | 332 | SATISFACTORY |
| 10 | 0 | 178 | SATISFACTORY |  | 10 | 1 | 1468 | SATISFACTORY |
| 10 | 0 | 32 | SATISFACTORY |  | 10 | 0 | 562 | SATISFACTORY |
| 10 | 0 | 178 | SATISFACTORY |  | 10 | 0 | 464 | SATISFACTORY |
| 10 | 1 | 351 | SATISFACTORY |  | 10 | 0 | 3162 | SATISFACTORY |
| 10 | 0 | 147 | SATISFACTORY |  | 10 | 0 | 316 | SATISFACTORY |
| 10 | 2 | 316 | SATISFACTORY |  | 10 | 0 | 3162 | SATISFACTORY |
| 10 | 1 | 351 | SATISFACTORY |  | 10 | 1 | 240 | SATISFACTORY |
| 10 | 0 | 646 | SATISFACTORY |  | 10 | 0 | 562 | SATISFACTORY |
| 10 | 0 | 1468 | SATISFACTORY |  | 10 | 0 | 3162 | SATISFACTORY |
| 10 | 0 | 316 | SATISFACTORY |  | 10 | 1 | 1000 | SATISFACTORY |
| 10 | 0 | 422 | SATISFACTORY |  | 10 | 0 | 422 | SATISFACTORY |
| 10 | 0 | 316 | SATISFACTORY |  | 10 | 2 | 10000 | SATISFACTORY |
| 10 | 1 | 4217 | SATISFACTORY |  | 10 | 0 | 3162 | SATISFACTORY |
| 10 | 0 | 351 | SATISFACTORY |  | 10 | 0 | 55 | SATISFACTORY |
| 10 | 0 | 562 | SATISFACTORY |  | 10 | 0 | 351 | SATISFACTORY |
| 10 | 0 | 215 | SATISFACTORY |  | 10 | 0 | 1778 | SATISFACTORY |
| 10 | 0 | 215 | SATISFACTORY |  | 10 | 0 | 562 | SATISFACTORY |
| 10 | 0 | 5012 | SATISFACTORY |  | 10 | 0 | 178 | SATISFACTORY |
| 10 | 0 | 178 | SATISFACTORY |  | 10 | 1 | 59 | SATISFACTORY |
| 10 | 0 | 562 | SATISFACTORY |  | 10 | 0 | 1995 | SATISFACTORY |
| 10 | 0 | 1000 | NO TEST |  | 10 | 0 | 3511 | SATISFACTORY |
| 10 | 1 | 481 | SATISFACTORY |  | 10 | 0 | 1778 | SATISFACTORY |
| 10 | 0 | 422 | SATISFACTORY |  | 10 | 1 | 1000 | SATISFACTORY |
| 10 | 0 | 1468 | SATISFACTORY |  | 10 | 0 | 676 | SATISFACTORY |
| 10 | 0 | 1778 | SATISFACTORY |  | 10 | 1 | 2371 | SATISFACTORY |
| 10 | 0 | 200 | SATISFACTORY |  | 10 | 2 | 3162 | SATISFACTORY |
| 10 | 1 | 32 | SATISFACTORY |  | 10 | 0 | 316 | SATISFACTORY |
| 10 | 0 | 3162 | SATISFACTORY |  | 10 | 0 | 150 | SATISFACTORY |
| 10 | 1 | 147 | SATISFACTORY |  | 10 | 0 | 1442 | SATISFACTORY |
| 10 | 0 | 228 | SATISFACTORY |  | 10 | 0 | 3162 | SATISFACTORY |
| 10 | 0 | 1000 | SATISFACTORY |  | 10 | 0 | 215 | SATISFACTORY |
| 10 | 0 | 1468 | SATISFACTORY |  | 10 | 1 | 178 | SATISFACTORY |
| 10 | 0 | 316 | SATISFACTORY |  | 10 | 0 | 237 | SATISFACTORY |
| 10 | 0 | 215 | SATISFACTORY |  | 10 | 0 | 794 | SATISFACTORY |
| 10 | 0 | 178 | SATISFACTORY |  | 10 | 2 | 2581 | SATISFACTORY |
| 10 | 0 | 178 | SATISFACTORY |  | 10 | 0 | 1442 | SATISFACTORY |
| 10 | 0 | 286 | SATISFACTORY |  | 10 | 0 | 46 | SATISFACTORY |
| 10 | 0 | 4786 | SATISFACTORY |  | 10 | 0 | 1778 | NO TEST |
| 10 | 0 | 178 | SATISFACTORY |  | 10 | 0 | 56 | SATISFACTORY |
| 10 | 5 | 6 | NO TEST |  | 10 | 0 | 1778 | SATISFACTORY |
| 10 | 0 | 316 | SATISFACTORY |  | 10 | 0 | 1468 | SATISFACTORY |
| 10 | 0 | 316 | SATISFACTORY |  | 10 | 0 | 1000 | SATISFACTORY |
| 10 | 0 | 200 | SATISFACTORY |  | 10 | 1 | 1000 | SATISFACTORY |
| 10 | 0 | 3162 | SATISFACTORY |  | 10 | 0 | 562 | SATISFACTORY |
| 10 | 2 | 1000 | SATISFACTORY |  | 10 | 0 | 1468 | SATISFACTORY |
| 10 | 1 | 100 | SATISFACTORY |  | 10 | 1 | 228 | SATISFACTORY |
| 10 | 0 | 562 | SATISFACTORY |  | 10 | 0 | 1778 | SATISFACTORY |
| 10 | 0 | 316 | Inconclusive |  | 10 | 0 | 562 | SATISFACTORY |
| 10 | 1 | 6310 | SATISFACTORY |  | 10 | 1 | 215 | SATISFACTORY |
| 10 | 1 | 100 | SATISFACTORY |  | 10 | 0 | 316 | SATISFACTORY |
| 10 | 0 | 562 | SATISFACTORY |  | 10 | 0 | 1778 | SATISFACTORY |
| 10 | 2 | 48 | SATISFACTORY |  | 10 | 0 | 562 | SATISFACTORY |
| 10 | 0 | 1655 | SATISFACTORY |  | 10 | 1 | 2154 | SATISFACTORY |
| 10 | 1 | 178 | SATISFACTORY |  | 10 | 0 | 398 | SATISFACTORY |
| 10 | 1 | 100 | SATISFACTORY |  | 10 | 1 | 501 | SATISFACTORY |
| 10 | 1 | 1000 | SATISFACTORY |  | 10 | 0 | 178 | SATISFACTORY |
| 10 | 0 | 10 | SATISFACTORY |  | 10 | 0 | 3455 | SATISFACTORY |
| 10 | 0 | 178 | SATISFACTORY |  | 10 | 0 | 794 | SATISFACTORY |
| 10 | 0 | 464 | SATISFACTORY |  | 10 | 0 | 562 | SATISFACTORY |
| 10 | 2 | 1000 | SATISFACTORY |  | 10 | 0 | 2154 | SATISFACTORY |
| 10 | 0 | 316 | SATISFACTORY |  | 10 | 0 | 316 | SATISFACTORY |
| 10 | 0 | 681 | SATISFACTORY |  | 10 | 1 | 1468 | SATISFACTORY |
| 10 | 0 | 562 | SATISFACTORY |  | 10 | 0 | 464 | SATISFACTORY |
| 10 | 0 | 215 | SATISFACTORY |  | 10 | 0 | 3162 | SATISFACTORY |
| 10 | 0 | 1000 | SATISFACTORY |  | 10 | 0 | 55 | SATISFACTORY |
| 10 | 2 | 316 | SATISFACTORY |  | 10 | 0 | 316 | SATISFACTORY |
| 10 | 0 | 203 | SATISFACTORY |  | 10 | 0 | 481 | SATISFACTORY |
| 10 | 0 | 562 | SATISFACTORY |  | 10 | 0 | 1794 | SATISFACTORY |
| 10 | 1 | 332 | SATISFACTORY |  | 10 | 0 | 3162 | SATISFACTORY |
| 10 | 0 | 1468 | SATISFACTORY |  | 10 | 0 | 1000 | SATISFACTORY |
| 10 | 0 | 316 | SATISFACTORY |  | 10 | 1 | 2154 | SATISFACTORY |
| 10 | 1 | 6310 | SATISFACTORY |  | 10 | 0 | 1000 | SATISFACTORY |
| 10 | 0 | 316 | SATISFACTORY |  | 10 | 0 | 1000 | SATISFACTORY |
| 10 | 0 | 316 | SATISFACTORY |  | 10 | 0 | 562 | SATISFACTORY |
| 10 | 1 | 422 | SATISFACTORY |  | 10 | 1 | 2371 | SATISFACTORY |
| 10 | 0 | 4786 | SATISFACTORY |  | 10 | 0 | 427 | SATISFACTORY |
| 10 | 0 | 32 | SATISFACTORY |  | 10 | 1 | 439 | SATISFACTORY |
| 10 | 1 | 42 | SATISFACTORY |  | 10 | 0 | 474 | SATISFACTORY |
| 10 | 0 | 200 | SATISFACTORY |  | 10 | 0 | 316 | SATISFACTORY |
| 10 | 0 | 681 | SATISFACTORY |  | 10 | 0 | 316 | SATISFACTORY |
| 10 | 1 | 468 | SATISFACTORY |  | 10 | 0 | 3162 | SATISFACTORY |
| 10 | 0 | 3162 | SATISFACTORY |  | 10 | 0 | 1778 | SATISFACTORY |
| 10 | 0 | 2344 | SATISFACTORY |  | 10 | 0 | 351 | SATISFACTORY |
| 10 | 0 | 4266 | SATISFACTORY |  | 10 | 1 | 1000 | SATISFACTORY |
| 10 | 0 | 75 | SATISFACTORY |  | 10 | 0 | 3162 | SATISFACTORY |
| 10 | 1 | 178 | SATISFACTORY |  | 10 | 0 | 3162 | SATISFACTORY |
| 10 | 0 | 316 | SATISFACTORY |  | 10 | 1 | 1000 | SATISFACTORY |
| 10 | 0 | 481 | SATISFACTORY |  | 10 | 0 | 147 | SATISFACTORY |
| 10 | 0 | 351 | SATISFACTORY |  | 10 | 0 | 316 | SATISFACTORY |
| 10 | 0 | 316 | SATISFACTORY |  | 10 | 0 | 3162 | SATISFACTORY |
| 10 | 1 | 178 | SATISFACTORY |  | 10 | 0 | 1778 | SATISFACTORY |
| 10 | 0 | 3162 | SATISFACTORY |  | 10 | 0 | 1995 | SATISFACTORY |
| 10 | 1 | 491 | SATISFACTORY |  | 10 | 2 | 178 | SATISFACTORY |
| 10 | 1 | 100 | SATISFACTORY |  | 10 | 0 | 1000 | SATISFACTORY |
| 10 | 0 | 178 | SATISFACTORY |  | 10 | 1 | 215 | SATISFACTORY |
| 10 | 0 | 200 | SATISFACTORY |  | 10 | 0 | 215 | SATISFACTORY |
| 10 | 3 | 20 | NO TEST |  | 10 | 0 | 178 | SATISFACTORY |
| 10 | 0 | 3162 | SATISFACTORY |  | 10 | 0 | 1778 | NO TEST |
| 10 | 0 | 3162 | SATISFACTORY |  | 10 | 1 | 1000 | SATISFACTORY |
| 10 | 1 | 351 | SATISFACTORY |  | 10 | 0 | 562 | SATISFACTORY |
| 10 | 2 | 1000 | SATISFACTORY |  | 10 | 1 | 346 | SATISFACTORY |
| 10 | 0 | 1000 | SATISFACTORY |  | 10 | 0 | 4217 | SATISFACTORY |
| 10 | 0 | 147 | SATISFACTORY |  | 10 | 1 | 631 | SATISFACTORY |
| 10 | 0 | 100 | SATISFACTORY |  | 10 | 0 | 1468 | SATISFACTORY |
| 10 | 1 | 1445 | SATISFACTORY |  | 10 | 0 | 316 | SATISFACTORY |
| 10 | 1 | 676 | SATISFACTORY |  | 10 | 0 | 316 | SATISFACTORY |
| 10 | 0 | 3162 | SATISFACTORY |  | 10 | 1 | 59 | SATISFACTORY |
| 10 | 0 | 464 | SATISFACTORY |  | 10 | 0 | 1468 | SATISFACTORY |
| 10 | 0 | 4169 | SATISFACTORY |  | 10 | 0 | 2512 | SATISFACTORY |
| 10 | 0 | 316 | SATISFACTORY |  | 10 | 0 | 351 | SATISFACTORY |
| 10 | 0 | 56 | SATISFACTORY |  | 10 | 1 | 1000 | SATISFACTORY |
| 10 | 0 | 316 | SATISFACTORY |  | 10 | 1 | 422 | SATISFACTORY |
| 10 | 0 | 2344 | SATISFACTORY |  | 10 | 0 | 316 | SATISFACTORY |
| 10 | 0 | 3162 | SATISFACTORY |  | 10 | 0 | 316 | SATISFACTORY |
| 10 | 1 | 1778 | SATISFACTORY |  | 10 | 0 | 562 | SATISFACTORY |
| 10 | 0 | 46 | SATISFACTORY |  | 10 | 0 | 215 | SATISFACTORY |
| 10 | 0 | 178 | SATISFACTORY |  | 10 | 2 | 126 | SATISFACTORY |
| 10 | 0 | 17783 | NO TEST |  | 10 | 0 | 1000 | SATISFACTORY |
| 10 | 1 | 3981 | SATISFACTORY |  | 10 | 0 | 332 | SATISFACTORY |
| 10 | 1 | 422 | SATISFACTORY |  | 10 | 0 | 562 | SATISFACTORY |
| 10 | 1 | 422 | SATISFACTORY |  | 10 | 0 | 1995 | SATISFACTORY |
| 10 | 0 | 501 | SATISFACTORY |  | 10 | 1 | 234 | SATISFACTORY |
| 10 | 0 | 10000 | SATISFACTORY |  | 10 | 0 | 2512 | SATISFACTORY |
| 10 | 0 | 624 | SATISFACTORY |  | 10 | 1 | 178 | SATISFACTORY |
| 10 | 1 | 1000 | SATISFACTORY |  | 10 | 0 | 170 | SATISFACTORY |
| 10 | 0 | 178 | SATISFACTORY |  | 10 | 0 | 3162 | SATISFACTORY |
| 10 | 0 | 200 | SATISFACTORY |  | 10 | 0 | 3162 | SATISFACTORY |
| 10 | 1 | 3162 | SATISFACTORY |  | 10 | 0 | 562 | SATISFACTORY |
| 10 | 1 | 178 | SATISFACTORY |  | 10 | 1 | 178 | SATISFACTORY |
| 10 | 0 | 389 | SATISFACTORY |  | 10 | 0 | 1422 | SATISFACTORY |
| 10 | 1 | 2154 | SATISFACTORY |  | 10 | 0 | 316 | SATISFACTORY |
| 10 | 1 | 250 | SATISFACTORY |  | 10 | 0 | 3162 | SATISFACTORY |
| 10 | 2 | 316 | SATISFACTORY |  | 10 | 1 | 422 | SATISFACTORY |
| 10 | 0 | 681 | SATISFACTORY |  | 10 | 0 | 3162 | SATISFACTORY |
| 10 | 1 | 346 | SATISFACTORY |  | 10 | 0 | 562 | SATISFACTORY |
| 10 | 0 | 478 | SATISFACTORY |  | 10 | 0 | 316 | SATISFACTORY |
| 10 | 2 | 10 | SATISFACTORY |  | 10 | 0 | 178 | SATISFACTORY |
| 10 | 1 | 2154 | SATISFACTORY |  | 10 | 0 | 1000 | SATISFACTORY |
| 10 | 0 | 2081 | SATISFACTORY |  | 10 | 1 | 1000 | SATISFACTORY |
| 10 | 0 | 316 | SATISFACTORY |  | 10 | 0 | 316 | SATISFACTORY |
| 10 | 0 | 3162 | SATISFACTORY |  | 10 | 0 | 1000 | SATISFACTORY |
| 10 | 0 | 178 | SATISFACTORY |  | 10 | 0 | 1202 | SATISFACTORY |
| 10 | 1 | 1000 | SATISFACTORY |  | 10 | 0 | 3162 | SATISFACTORY |
| 10 | 0 | 10 | SATISFACTORY |  | 10 | 0 | 631 | SATISFACTORY |
| 10 | 0 | 178 | SATISFACTORY |  | 10 | 0 | 562 | SATISFACTORY |
| 10 | 1 | 32 | SATISFACTORY |  | 10 | 0 | 1778 | NO TEST |
| 10 | 0 | 1000 | SATISFACTORY |  | 10 | 0 | 562 | SATISFACTORY |
| 10 | 0 | 562 | SATISFACTORY |  | 10 | 0 | 1468 | SATISFACTORY |
| 10 | 0 | 3162 | SATISFACTORY |  | 10 | 0 | 562 | SATISFACTORY |
| 10 | 1 | 3162 | SATISFACTORY |  | 10 | 0 | 4806 | SATISFACTORY |
| 10 | 0 | 3162 | SATISFACTORY |  | 10 | 0 | 1468 | SATISFACTORY |
| 10 | 0 | 10 | SATISFACTORY |  | 10 | 0 | 1778 | SATISFACTORY |
| 10 | 0 | 56 | SATISFACTORY |  | 10 | 0 | 316 | SATISFACTORY |
| 10 | 0 | 427 | SATISFACTORY |  | 10 | 0 | 6761 | SATISFACTORY |
| 10 | 0 | 5012 | SATISFACTORY |  | 10 | 0 | 316 | SATISFACTORY |
| 10 | 0 | 3162 | SATISFACTORY |  | 10 | 0 | 351 | SATISFACTORY |
| 10 | 0 | 316 | SATISFACTORY |  | 10 | 1 | 228 | SATISFACTORY |
| 10 | 1 | 427 | SATISFACTORY |  | 10 | 0 | 215 | SATISFACTORY |
| 10 | 0 | 3162 | SATISFACTORY |  | 10 | 0 | 6812 | SATISFACTORY |
| 10 | 1 | 100 | SATISFACTORY |  | 10 | 0 | 1778 | SATISFACTORY |
| 10 | 1 | 422 | SATISFACTORY |  | 10 | 1 | 599 | SATISFACTORY |
| 10 | 1 | 351 | SATISFACTORY |  | 10 | 0 | 562 | SATISFACTORY |
| 10 | 0 | 631 | SATISFACTORY |  | 10 | 0 | 1000 | SATISFACTORY |
| 10 | 0 | 316 | SATISFACTORY |  | 10 | 0 | 3162 | SATISFACTORY |
| 10 | 0 | 10000 | SATISFACTORY |  | 10 | 1 | 178 | SATISFACTORY |
| 10 | 1 | 422 | SATISFACTORY |  | 10 | 2 | 10000 | SATISFACTORY |
| 10 | 2 | 32 | SATISFACTORY |  | 10 | 0 | 4217 | SATISFACTORY |
| 10 | 0 | 228 | SATISFACTORY |  | 10 | 0 | 215 | SATISFACTORY |
| 10 | 0 | 178 | SATISFACTORY |  | 10 | 0 | 1000 | SATISFACTORY |
| 10 | 1 | 14791 | NO TEST |  | 10 | 1 | 1000 | SATISFACTORY |
| 10 | 0 | 215 | SATISFACTORY |  | 10 | 0 | 3162 | SATISFACTORY |
| 10 | 0 | 10000 | SATISFACTORY |  | 10 | 1 | 178 | SATISFACTORY |
| 10 | 1 | 692 | SATISFACTORY |  | 10 | 0 | 316 | SATISFACTORY |
| 10 | 0 | 79 | SATISFACTORY |  | 10 | 0 | 562 | SATISFACTORY |
| 10 | 0 | 562 | SATISFACTORY |  | 10 | 0 | 1000 | SATISFACTORY |
| 10 | 0 | 1000 | SATISFACTORY |  | 10 | 0 | 59 | SATISFACTORY |
| 10 | 0 | 316 | SATISFACTORY |  | 10 | 0 | 316 | SATISFACTORY |
| 10 | 0 | 316 | SATISFACTORY |  | 10 | 0 | 5623 | SATISFACTORY |
| 10 | 0 | 1000 | SATISFACTORY |  | 10 | 0 | 562 | SATISFACTORY |
| 10 | 0 | 624 | SATISFACTORY |  | 10 | 0 | 5623 | SATISFACTORY |
| 10 | 0 | 1000 | SATISFACTORY |  | 10 | 0 | 351 | SATISFACTORY |
| 10 | 0 | 422 | SATISFACTORY |  | 10 | 1 | 178 | SATISFACTORY |
| 10 | 1 | 422 | SATISFACTORY |  | 10 | 1 | 234 | SATISFACTORY |
| 10 | 0 | 464 | SATISFACTORY |  | 10 | 1 | 422 | SATISFACTORY |
| 10 | 0 | 316 | SATISFACTORY |  | 10 | 0 | 3162 | SATISFACTORY |
| 10 | 0 | 624 | Inconclusive |  | 10 | 1 | 1000 | SATISFACTORY |
| 10 | 1 | 151 | SATISFACTORY |  | 10 | 0 | 1585 | SATISFACTORY |
| 10 | 1 | 215 | SATISFACTORY |  | 10 | 1 | 1000 | SATISFACTORY |
| 10 | 0 | 1468 | SATISFACTORY |  | 10 | 0 | 427 | SATISFACTORY |
| 10 | 1 | 42 | SATISFACTORY |  | 10 | 0 | 562 | SATISFACTORY |
| 10 | 1 | 676 | SATISFACTORY |  | 10 | 0 | 316 | SATISFACTORY |
| 10 | 0 | 1778 | SATISFACTORY |  | 10 | 0 | 427 | SATISFACTORY |
| 10 | 0 | 10001 | NO TEST |  | 10 | 0 | 562 | SATISFACTORY |
| 10 | 1 | 147 | SATISFACTORY |  | 10 | 0 | 1000 | SATISFACTORY |
| 10 | 0 | 316 | SATISFACTORY |  | 10 | 0 | 562 | SATISFACTORY |
| 10 | 1 | 2154 | SATISFACTORY |  | 10 | 0 | 3162 | SATISFACTORY |
| 10 | 0 | 2512 | SATISFACTORY |  | 10 | 1 | 346 | SATISFACTORY |
| 10 | 0 | 2371 | SATISFACTORY |  | 10 | 0 | 3455 | SATISFACTORY |
| 10 | 1 | 422 | SATISFACTORY |  | 10 | 1 | 351 | SATISFACTORY |
| 10 | 1 | 351 | SATISFACTORY |  | 10 | 1 | 316 | SATISFACTORY |
| 10 | 0 | 178 | SATISFACTORY |  | 10 | 1 | 1000 | SATISFACTORY |
| 10 | 0 | 427 | SATISFACTORY |  | 10 | 1 | 1000 | SATISFACTORY |
| 10 | 0 | 681 | SATISFACTORY |  | 10 | 0 | 170 | SATISFACTORY |
| 10 | 0 | 178 | SATISFACTORY |  | 10 | 0 | 214 | SATISFACTORY |
| 10 | 2 | 2081 | SATISFACTORY |  | 10 | 0 | 681 | SATISFACTORY |
| 10 | 1 | 100 | SATISFACTORY |  | 10 | 0 | 3162 | SATISFACTORY |
| 10 | 2 | 100 | SATISFACTORY |  | 10 | 0 | 562 | SATISFACTORY |
| 10 | 0 | 3162 | SATISFACTORY |  | 10 | 0 | 3162 | SATISFACTORY |
| 10 | 0 | 234 | SATISFACTORY |  | 10 | 0 | 562 | SATISFACTORY |
| 10 | 1 | 42 | SATISFACTORY |  | 10 | 1 | 59 | SATISFACTORY |
| 10 | 0 | 5623 | SATISFACTORY |  | 10 | 1 | 59 | SATISFACTORY |
| 10 | 0 | 1468 | SATISFACTORY |  | 10 | 0 | 316 | SATISFACTORY |
| 10 | 0 | 562 | SATISFACTORY |  | 10 | 0 | 3162 | SATISFACTORY |
| 10 | 0 | 316 | SATISFACTORY |  | 10 | 1 | 1000 | SATISFACTORY |
| 10 | 1 | 1778 | SATISFACTORY |  | 10 | 0 | 1445 | SATISFACTORY |
| 10 | 0 | 3162 | SATISFACTORY |  | 10 | 0 | 5623 | SATISFACTORY |
| 10 | 0 | 215 | SATISFACTORY |  | 10 | 0 | 588 | SATISFACTORY |
| 10 | 0 | 3495 | SATISFACTORY |  | 10 | 0 | 1000 | SATISFACTORY |
| 10 | 0 | 316 | SATISFACTORY |  | 10 | 1 | 2081 | SATISFACTORY |
| 10 | 2 | 1000 | SATISFACTORY |  | 10 | 1 | 179 | SATISFACTORY |
| 10 | 1 | 147 | SATISFACTORY |  | 10 | 0 | 562 | SATISFACTORY |
| 10 | 0 | 389 | SATISFACTORY |  | 10 | 1 | 178 | SATISFACTORY |
| 10 | 0 | 3162 | SATISFACTORY |  | 10 | 1 | 481 | SATISFACTORY |
| 10 | 0 | 422 | SATISFACTORY |  | 10 | 1 | 562 | SATISFACTORY |
| 10 | 0 | 18 | SATISFACTORY |  | 10 | 1 | 178 | SATISFACTORY |
| 10 | 1 | 100 | SATISFACTORY |  | 10 | 0 | 3162 | SATISFACTORY |
| 10 | 0 | 1468 | SATISFACTORY |  | 10 | 0 | 351 | SATISFACTORY |
| 10 | 1 | 100 | SATISFACTORY |  | 10 | 0 | 1000 | SATISFACTORY |
| 10 | 2 | 63 | SATISFACTORY |  | 10 | 0 | 3162 | SATISFACTORY |
| 10 | 1 | 1468 | SATISFACTORY |  | 10 | 0 | 316 | SATISFACTORY |
| 10 | 0 | 2081 | SATISFACTORY |  | 10 | 0 | 3162 | SATISFACTORY |
| 10 | 0 | 422 | SATISFACTORY |  | 10 | 0 | 2154 | SATISFACTORY |
| 10 | 0 | 40 | SATISFACTORY |  | 10 | 0 | 3162 | SATISFACTORY |
| 10 | 1 | 178 | SATISFACTORY |  | 10 | 0 | 2371 | SATISFACTORY |
| 10 | 3 | 2 | NO TEST |  | 10 | 0 | 562 | SATISFACTORY |
| 10 | 0 | 10000 | SATISFACTORY |  | 10 | 0 | 562 | SATISFACTORY |
| 10 | 0 | 1995 | SATISFACTORY |  | 10 | 0 | 1259 | SATISFACTORY |
| 10 | 0 | 3162 | SATISFACTORY |  | 10 | 0 | 150 | SATISFACTORY |
| 10 | 2 | 1000 | SATISFACTORY |  | 10 | 1 | 59 | SATISFACTORY |
| 10 | 1 | 422 | SATISFACTORY |  | 10 | 1 | 1000 | SATISFACTORY |
| 10 | 0 | 5495 | SATISFACTORY |  | 10 | 1 | 1995 | SATISFACTORY |
| 10 | 0 | 558 | SATISFACTORY |  | 10 | 1 | 422 | SATISFACTORY |
| 10 | 0 | 562 | SATISFACTORY |  | 10 | 1 | 1000 | SATISFACTORY |
| 10 | 1 | 351 | SATISFACTORY |  | 10 | 1 | 215 | SATISFACTORY |
| 10 | 0 | 215 | SATISFACTORY |  | 10 | 1 | 1000 | SATISFACTORY |
| 10 | 0 | 215 | SATISFACTORY |  | 10 | 1 | 351 | SATISFACTORY |
| 10 | 0 | 316 | SATISFACTORY |  | 10 | 0 | 332 | SATISFACTORY |
| 10 | 0 | 2081 | SATISFACTORY |  | 10 | 0 | 1000 | SATISFACTORY |
| 10 | 1 | 1000 | SATISFACTORY |  | 10 | 0 | 316 | SATISFACTORY |
| 10 | 1 | 1468 | SATISFACTORY |  | 10 | 0 | 316 | SATISFACTORY |
| 10 | 0 | 316 | SATISFACTORY |  | 10 | 0 | 200 | SATISFACTORY |
| 10 | 0 | 3162 | SATISFACTORY |  | 10 | 0 | 170 | SATISFACTORY |
| 10 | 0 | 178 | SATISFACTORY |  | 10 | 0 | 1778 | SATISFACTORY |
| 10 | 1 | 1468 | SATISFACTORY |  | 10 | 0 | 1778 | SATISFACTORY |
| 10 | 0 | 681 | SATISFACTORY |  | 10 | 0 | 316 | SATISFACTORY |
| 10 | 1 | 100 | SATISFACTORY |  | 10 | 0 | 1738 | SATISFACTORY |
| 10 | 0 | 681 | SATISFACTORY |  | 10 | 1 | 1000 | SATISFACTORY |
| 10 | 0 | 316 | SATISFACTORY |  | 10 | 0 | 178 | SATISFACTORY |
| 10 | 0 | 228 | SATISFACTORY |  | 10 | 0 | 2093 | SATISFACTORY |
| 10 | 0 | 550 | SATISFACTORY |  | 10 | 0 | 3511 | SATISFACTORY |
| 10 | 0 | 209 | SATISFACTORY |  | 10 | 0 | 588 | SATISFACTORY |
| 10 | 0 | 10000 | SATISFACTORY |  | 10 | 0 | 316 | SATISFACTORY |
| 10 | 1 | 481 | SATISFACTORY |  | 10 | 0 | 316 | SATISFACTORY |
| 10 | 1 | 100 | SATISFACTORY |  | 10 | 0 | 1442 | SATISFACTORY |
| 10 | 0 | 351 | SATISFACTORY |  | 10 | 1 | 1000 | SATISFACTORY |
| 10 | 1 | 178 | SATISFACTORY |  | 10 | 0 | 2154 | SATISFACTORY |
| 10 | 0 | 562 | SATISFACTORY |  | 10 | 0 | 492 | SATISFACTORY |
| 10 | 1 | 351 | SATISFACTORY |  | 10 | 0 | 3162 | SATISFACTORY |
| 10 | 0 | 32 | SATISFACTORY |  | 10 | 1 | 178 | SATISFACTORY |
| 10 | 0 | 228 | SATISFACTORY |  | 10 | 0 | 351 | SATISFACTORY |
| 10 | 0 | 178 | SATISFACTORY |  | 10 | 0 | 208 | SATISFACTORY |
| 10 | 1 | 100 | SATISFACTORY |  | 10 | 0 | 3162 | SATISFACTORY |
| 10 | 1 | 1000 | SATISFACTORY |  | 10 | 0 | 65 | SATISFACTORY |
| 10 | 0 | 562 | SATISFACTORY |  | 10 | 1 | 178 | SATISFACTORY |
| 10 | 0 | 501 | SATISFACTORY |  | 10 | 0 | 3162 | SATISFACTORY |
| 10 | 0 | 234 | SATISFACTORY |  | 10 | 0 | 215 | SATISFACTORY |
| 10 | 0 | 17783 | NO TEST |  | 10 | 0 | 5623 | SATISFACTORY |
| 10 | 0 | 234 | SATISFACTORY |  | 10 | 0 | 1778 | SATISFACTORY |
| 10 | 0 | 316 | SATISFACTORY |  | 10 | 0 | 1778 | SATISFACTORY |
| 10 | 1 | 422 | SATISFACTORY |  | 10 | 0 | 3162 | SATISFACTORY |
| 10 | 0 | 2344 | SATISFACTORY |  | 10 | 0 | 676 | SATISFACTORY |
| 10 | 0 | 3162 | SATISFACTORY |  | 10 | 0 | 427 | SATISFACTORY |
| 10 | 0 | 316 | SATISFACTORY |  | 10 | 1 | 631 | SATISFACTORY |
| 10 | 1 | 35 | SATISFACTORY |  | 10 | 0 | 562 | SATISFACTORY |
| 10 | 0 | 1000 | SATISFACTORY |  | 10 | 0 | 3162 | SATISFACTORY |
| 10 | 0 | 1468 | SATISFACTORY |  | 10 | 0 | 1000 | SATISFACTORY |
| 10 | 0 | 228 | SATISFACTORY |  | 10 | 0 | 178 | SATISFACTORY |
| 10 | 0 | 3162 | SATISFACTORY |  | 10 | 0 | 3162 | SATISFACTORY |
| 10 | 0 | 6 | NO TEST |  | 10 | 0 | 3455 | SATISFACTORY |
| 10 | 0 | 316 | SATISFACTORY |  | 10 | 0 | 316 | SATISFACTORY |
| 10 | 0 | 200 | SATISFACTORY |  | 10 | 0 | 2276 | SATISFACTORY |
| 10 | 0 | 681 | SATISFACTORY |  | 10 | 2 | 228 | SATISFACTORY |
| 10 | 0 | 478 | SATISFACTORY |  | 10 | 0 | 562 | SATISFACTORY |
| 10 | 1 | 215 | SATISFACTORY |  | 10 | 0 | 1349 | SATISFACTORY |
| 10 | 0 | 422 | SATISFACTORY |  | 10 | 1 | 1000 | SATISFACTORY |
| 10 | 0 | 316 | SATISFACTORY |  | 10 | 2 | 316 | SATISFACTORY |
| 10 | 1 | 215 | SATISFACTORY |  | 10 | 0 | 316 | SATISFACTORY |
| 10 | 1 | 1000 | SATISFACTORY |  | 10 | 0 | 1000 | SATISFACTORY |
| 10 | 0 | 10000 | SATISFACTORY |  | 10 | 0 | 681 | SATISFACTORY |
| 10 | 1 | 1000 | SATISFACTORY |  | 10 | 1 | 422 | SATISFACTORY |
| 10 | 0 | 3162 | SATISFACTORY |  | 10 | 0 | 1778 | SATISFACTORY |
| 10 | 1 | 1778 | SATISFACTORY |  | 10 | 0 | 351 | SATISFACTORY |
| 10 | 1 | 351 | SATISFACTORY |  | 10 | 1 | 422 | SATISFACTORY |
| 10 | 1 | 1000 | SATISFACTORY |  | 10 | 0 | 3162 | SATISFACTORY |
| 10 | 1 | 351 | SATISFACTORY |  | 10 | 1 | 165 | SATISFACTORY |
| 10 | 0 | 562 | SATISFACTORY |  | 10 | 0 | 200 | SATISFACTORY |
| 10 | 1 | 100 | SATISFACTORY |  | 10 | 1 | 234 | SATISFACTORY |
| 10 | 0 | 316 | SATISFACTORY |  | 10 | 1 | 165 | SATISFACTORY |
| 10 | 0 | 1468 | SATISFACTORY |  | 10 | 0 | 2371 | SATISFACTORY |
| 10 | 0 | 240 | SATISFACTORY |  | 10 | 0 | 464 | SATISFACTORY |
| 10 | 0 | 10000 | SATISFACTORY |  | 10 | 0 | 170 | SATISFACTORY |
| 10 | 1 | 351 | SATISFACTORY |  | 10 | 1 | 681 | SATISFACTORY |
| 10 | 0 | 316 | SATISFACTORY |  | 10 | 0 | 24 | SATISFACTORY |
| 10 | 1 | 1000 | SATISFACTORY |  | 10 | 0 | 2154 | SATISFACTORY |
| 10 | 0 | 178 | SATISFACTORY |  | 10 | 0 | 316 | SATISFACTORY |
| 10 | 0 | 422 | SATISFACTORY |  | 10 | 1 | 1995 | SATISFACTORY |
| 10 | 1 | 351 | SATISFACTORY |  | 10 | 1 | 346 | SATISFACTORY |
| 10 | 0 | 237 | SATISFACTORY |  | 10 | 0 | 100 | SATISFACTORY |
| 10 | 0 | 480 | SATISFACTORY |  | 10 | 0 | 316 | SATISFACTORY |
| 10 | 1 | 50 | SATISFACTORY |  | 10 | 0 | 316 | SATISFACTORY |
| 10 | 0 | 17783 | NO TEST |  | 10 | 0 | 562 | SATISFACTORY |
| 10 | 0 | 316 | SATISFACTORY |  | 10 | 0 | 316 | SATISFACTORY |
| 10 | 1 | 14791 | NO TEST |  | 10 | 0 | 316 | SATISFACTORY |
| 10 | 0 | 1000 | SATISFACTORY |  | 10 | 1 | 1000 | SATISFACTORY |
| 10 | 1 | 422 | SATISFACTORY |  | 10 | 1 | 178 | SATISFACTORY |
| 10 | 0 | 228 | SATISFACTORY |  | 10 | 0 | 1000 | SATISFACTORY |
| 10 | 1 | 481 | SATISFACTORY |  | 10 | 0 | 1778 | SATISFACTORY |
| 10 | 0 | 1468 | SATISFACTORY |  | 10 | 1 | 1000 | SATISFACTORY |
| 10 | 0 | 4786 | SATISFACTORY |  | 10 | 0 | 316 | SATISFACTORY |
| 10 | 0 | 1468 | SATISFACTORY |  | 10 | 0 | 1000 | SATISFACTORY |
| 10 | 2 | 1000 | SATISFACTORY |  | 10 | 0 | 562 | SATISFACTORY |
| 10 | 0 | 562 | SATISFACTORY |  | 10 | 0 | 3162 | SATISFACTORY |
| 10 | 0 | 562 | SATISFACTORY |  | 10 | 0 | 316 | SATISFACTORY |
| 10 | 1 | 18 | SATISFACTORY |  | 10 | 0 | 170 | SATISFACTORY |
| 10 | 0 | 422 | SATISFACTORY |  | 10 | 1 | 439 | SATISFACTORY |
| 10 | 0 | 316 | SATISFACTORY |  | 10 | 2 | 7244 | SATISFACTORY |
| 10 | 0 | 126 | SATISFACTORY |  | 10 | 0 | 316 | SATISFACTORY |
| 10 | 0 | 422 | SATISFACTORY |  | 10 | 0 | 1000 | SATISFACTORY |
| 10 | 1 | 215 | SATISFACTORY |  | 10 | 0 | 3511 | SATISFACTORY |
| 10 | 0 | 1000 | SATISFACTORY |  | 10 | 0 | 562 | SATISFACTORY |
| 10 | 0 | 10001 | NO TEST |  | 10 | 1 | 351 | SATISFACTORY |
| 10 | 1 | 147 | SATISFACTORY |  | 10 | 0 | 215 | SATISFACTORY |
| 10 | 0 | 692 | SATISFACTORY |  | 10 | 0 | 1000 | SATISFACTORY |
| 10 | 0 | 316 | SATISFACTORY |  | 10 | 0 | 178 | SATISFACTORY |
| 10 | 0 | 32 | SATISFACTORY |  | 10 | 0 | 6310 | SATISFACTORY |
| 10 | 0 | 1000 | SATISFACTORY |  | 10 | 1 | 178 | SATISFACTORY |
| 10 | 0 | 562 | SATISFACTORY |  | 10 | 0 | 316 | SATISFACTORY |
| 10 | 0 | 316 | SATISFACTORY |  | 10 | 0 | 3511 | SATISFACTORY |
| 10 | 0 | 178 | SATISFACTORY |  | 10 | 1 | 439 | SATISFACTORY |
| 10 | 1 | 159 | SATISFACTORY |  | 10 | 1 | 178 | SATISFACTORY |
| 10 | 0 | 681 | SATISFACTORY |  | 10 | 0 | 3162 | SATISFACTORY |
| 10 | 1 | 1778 | SATISFACTORY |  | 10 | 1 | 489 | SATISFACTORY |
| 10 | 0 | 228 | SATISFACTORY |  | 10 | 0 | 2371 | SATISFACTORY |
| 10 | 0 | 32 | SATISFACTORY |  | 10 | 0 | 794 | SATISFACTORY |
| 10 | 0 | 562 | SATISFACTORY |  | 10 | 0 | 2371 | SATISFACTORY |
| 10 | 1 | 351 | SATISFACTORY |  | 10 | 0 | 214 | SATISFACTORY |
| 10 | 1 | 100 | SATISFACTORY |  | 10 | 0 | 1778 | SATISFACTORY |
| 10 | 0 | 178 | SATISFACTORY |  | 10 | 0 | 1000 | SATISFACTORY |
| 10 | 0 | 2089 | SATISFACTORY |  | 10 | 0 | 215 | SATISFACTORY |
| 10 | 0 | 427 | SATISFACTORY |  | 10 | 0 | 3162 | SATISFACTORY |
| 10 | 0 | 624 | SATISFACTORY |  | 10 | 0 | 562 | SATISFACTORY |
| 10 | 0 | 346 | SATISFACTORY |  | 10 | 0 | 3162 | SATISFACTORY |
| 10 | 1 | 32 | SATISFACTORY |  | 10 | 0 | 3511 | SATISFACTORY |
| 10 | 0 | 10 | SATISFACTORY |  | 10 | 0 | 3162 | SATISFACTORY |
| 10 | 0 | 3162 | SATISFACTORY |  | 10 | 0 | 316 | SATISFACTORY |
| 10 | 0 | 562 | SATISFACTORY |  | 10 | 1 | 1000 | SATISFACTORY |
| 10 | 0 | 215 | SATISFACTORY |  | 10 | 0 | 562 | SATISFACTORY |
| 10 | 0 | 478 | SATISFACTORY |  | 10 | 0 | 1000 | SATISFACTORY |
| 10 | 0 | 1778 | SATISFACTORY |  | 10 | 0 | 3162 | SATISFACTORY |
| 10 | 0 | 316 | SATISFACTORY |  | 10 | 0 | 1000 | SATISFACTORY |
| 10 | 0 | 316 | SATISFACTORY |  | 10 | 0 | 178 | SATISFACTORY |
| 10 | 1 | 351 | SATISFACTORY |  | 10 | 0 | 562 | SATISFACTORY |
| 10 | 1 | 215 | SATISFACTORY |  | 10 | 0 | 182 | SATISFACTORY |
| 10 | 0 | 208 | SATISFACTORY |  | 10 | 0 | 562 | SATISFACTORY |
| 10 | 1 | 1000 | SATISFACTORY |  | 10 | 0 | 3162 | SATISFACTORY |
| 10 | 0 | 4266 | SATISFACTORY |  | 10 | 1 | 63 | SATISFACTORY |
| 10 | 0 | 2344 | SATISFACTORY |  | 10 | 0 | 794 | SATISFACTORY |
| 10 | 0 | 10000 | SATISFACTORY |  | 10 | 0 | 562 | SATISFACTORY |
| 10 | 2 | 23 | SATISFACTORY |  | 10 | 1 | 631 | SATISFACTORY |
| 10 | 0 | 422 | SATISFACTORY |  | 10 | 0 | 1000 | SATISFACTORY |
| 10 | 0 | 237 | SATISFACTORY |  | 10 | 0 | 3511 | SATISFACTORY |
| 10 | 1 | 178 | SATISFACTORY |  | 10 | 0 | 4217 | SATISFACTORY |
| 10 | 0 | 1000 | SATISFACTORY |  | 10 | 1 | 427 | SATISFACTORY |
| 10 | 0 | 1995 | SATISFACTORY |  | 10 | 0 | 1000 | SATISFACTORY |
| 10 | 0 | 692 | SATISFACTORY |  | 10 | 0 | 4217 | SATISFACTORY |
| 10 | 0 | 316 | SATISFACTORY |  | 10 | 1 | 178 | SATISFACTORY |
| 10 | 0 | 1468 | SATISFACTORY |  | 10 | 1 | 240 | SATISFACTORY |
| 10 | 0 | 178 | SATISFACTORY |  | 10 | 0 | 562 | SATISFACTORY |
| 10 | 0 | 75 | SATISFACTORY |  | 10 | 0 | 237 | SATISFACTORY |
| 10 | 2 | 15 | SATISFACTORY |  | 10 | 0 | 3162 | SATISFACTORY |
| 10 | 0 | 1000 | SATISFACTORY |  | 10 | 0 | 3162 | SATISFACTORY |
| 10 | 0 | 562 | SATISFACTORY |  | 10 | 0 | 491 | SATISFACTORY |
| 10 | 1 | 42 | SATISFACTORY |  | 10 | 1 | 215 | SATISFACTORY |
| 10 | 1 | 178 | SATISFACTORY |  | 10 | 0 | 3162 | SATISFACTORY |
| 10 | 1 | 386 | SATISFACTORY |  | 10 | 0 | 351 | SATISFACTORY |
| 10 | 0 | 422 | SATISFACTORY |  | 10 | 0 | 2093 | SATISFACTORY |
| 10 | 0 | 1000 | SATISFACTORY |  | 10 | 0 | 237 | SATISFACTORY |
| 10 | 1 | 1479 | SATISFACTORY |  | 10 | 0 | 3162 | SATISFACTORY |
| 10 | 0 | 200 | SATISFACTORY |  | 10 | 0 | 50 | SATISFACTORY |
| 10 | 0 | 316 | SATISFACTORY |  | 10 | 1 | 351 | SATISFACTORY |
| 10 | 0 | 18 | SATISFACTORY |  | 10 | 1 | 3325 | SATISFACTORY |
| 10 | 0 | 2081 | SATISFACTORY |  | 10 | 0 | 178 | SATISFACTORY |
| 10 | 0 | 209 | SATISFACTORY |  | 10 | 1 | 215 | SATISFACTORY |
| 10 | 0 | 1468 | SATISFACTORY |  | 10 | 0 | 562 | SATISFACTORY |
| 10 | 0 | 50 | SATISFACTORY |  | 10 | 1 | 1000 | SATISFACTORY |
| 10 | 1 | 692 | SATISFACTORY |  | 10 | 1 | 1000 | SATISFACTORY |
| 10 | 0 | 491 | SATISFACTORY |  | 10 | 0 | 2512 | Inconclusive |
| 10 | 0 | 316 | SATISFACTORY |  | 10 | 1 | 59 | SATISFACTORY |
| 10 | 1 | 422 | SATISFACTORY |  | 10 | 0 | 1000 | SATISFACTORY |
| 10 | 0 | 1479 | SATISFACTORY |  | 10 | 0 | 1096 | SATISFACTORY |
| 10 | 1 | 386 | SATISFACTORY |  | 10 | 0 | 1000 | SATISFACTORY |
| 10 | 1 | 178 | SATISFACTORY |  | 10 | 0 | 215 | SATISFACTORY |
| 10 | 0 | 178 | SATISFACTORY |  | 10 | 0 | 2371 | SATISFACTORY |
| 10 | 0 | 75 | SATISFACTORY |  | 10 | 0 | 3162 | SATISFACTORY |
| 10 | 0 | 55 | SATISFACTORY |  | 10 | 0 | 550 | SATISFACTORY |
| 10 | 1 | 178 | SATISFACTORY |  | 10 | 0 | 603 | SATISFACTORY |
| 10 | 1 | 422 | SATISFACTORY |  | 10 | 0 | 3162 | SATISFACTORY |
| 10 | 1 | 351 | SATISFACTORY |  | 10 | 0 | 3511 | SATISFACTORY |
| 10 | 0 | 562 | SATISFACTORY |  | 10 | 0 | 676 | SATISFACTORY |
| 10 | 0 | 316 | SATISFACTORY |  | 10 | 1 | 700 | SATISFACTORY |
| 10 | 0 | 178 | SATISFACTORY |  | 10 | 0 | 56 | SATISFACTORY |
| 10 | 0 | 3162 | SATISFACTORY |  | 10 | 0 | 1000 | SATISFACTORY |
| 10 | 0 | 1479 | SATISFACTORY |  | 10 | 1 | 178 | SATISFACTORY |
| 10 | 1 | 422 | SATISFACTORY |  | 10 | 1 | 178 | SATISFACTORY |
| 10 | 0 | 5623 | SATISFACTORY |  | 10 | 0 | 10000 | SATISFACTORY |
| 10 | 0 | 316 | SATISFACTORY |  | 10 | 0 | 562 | SATISFACTORY |
| 10 | 0 | 398 | SATISFACTORY |  | 10 | 1 | 1000 | SATISFACTORY |
| 10 | 0 | 501 | SATISFACTORY |  | 10 | 0 | 2093 | SATISFACTORY |
| 10 | 1 | 100 | SATISFACTORY |  | 10 | 0 | 178 | SATISFACTORY |
| 10 | 2 | 25 | SATISFACTORY |  | 10 | 0 | 316 | SATISFACTORY |
| 10 | 0 | 692 | SATISFACTORY |  | 10 | 1 | 1000 | SATISFACTORY |
| 10 | 2 | 48 | SATISFACTORY |  | 10 | 1 | 178 | SATISFACTORY |
| 10 | 1 | 422 | SATISFACTORY |  | 10 | 0 | 50 | SATISFACTORY |
| 10 | 0 | 3162 | SATISFACTORY |  | 10 | 0 | 1000 | SATISFACTORY |
| 10 | 0 | 316 | SATISFACTORY |  | 10 | 1 | 178 | SATISFACTORY |
| 10 | 0 | 1000 | SATISFACTORY |  | 10 | 2 | 182 | SATISFACTORY |
| 10 | 0 | 1000 | SATISFACTORY |  | 10 | 0 | 316 | SATISFACTORY |
| 10 | 1 | 422 | SATISFACTORY |  | 10 | 0 | 955 | SATISFACTORY |
| 10 | 0 | 215 | SATISFACTORY |  | 10 | 0 | 1585 | SATISFACTORY |
| 10 | 0 | 1000 | SATISFACTORY |  | 10 | 0 | 178 | SATISFACTORY |
| 10 | 2 | 316 | SATISFACTORY |  | 10 | 1 | 1468 | SATISFACTORY |
| 10 | 0 | 3162 | SATISFACTORY |  | 10 | 1 | 240 | SATISFACTORY |
| 10 | 1 | 32 | SATISFACTORY |  | 10 | 1 | 1995 | SATISFACTORY |
| 10 | 0 | 32 | SATISFACTORY |  | 10 | 1 | 1000 | SATISFACTORY |
| 10 | 0 | 10000 | SATISFACTORY |  | 10 | 1 | 178 | SATISFACTORY |
| 10 | 1 | 178 | SATISFACTORY |  | 10 | 1 | 501 | SATISFACTORY |
| 10 | 1 | 208 | SATISFACTORY |  | 10 | 0 | 3511 | SATISFACTORY |
| 10 | 0 | 562 | SATISFACTORY |  | 10 | 0 | 1468 | SATISFACTORY |
| 10 | 0 | 178 | SATISFACTORY |  | 10 | 0 | 1778 | SATISFACTORY |
| 10 | 1 | 100 | SATISFACTORY |  | 10 | 0 | 562 | SATISFACTORY |
| 10 | 0 | 215 | SATISFACTORY |  | 10 | 0 | 1445 | SATISFACTORY |
| 10 | 1 | 386 | SATISFACTORY |  | 10 | 0 | 422 | SATISFACTORY |
| 10 | 0 | 1468 | SATISFACTORY |  | 10 | 1 | 228 | SATISFACTORY |
| 10 | 0 | 179 | SATISFACTORY |  | 10 | 0 | 100 | SATISFACTORY |
| 10 | 0 | 562 | SATISFACTORY |  | 10 | 0 | 316 | SATISFACTORY |
| 10 | 1 | 179 | SATISFACTORY |  | 10 | 0 | 1468 | SATISFACTORY |
| 10 | 0 | 316 | SATISFACTORY |  | 10 | 0 | 3162 | SATISFACTORY |
| 10 | 0 | 562 | SATISFACTORY |  | 10 | 1 | 59 | SATISFACTORY |
| 10 | 0 | 3162 | SATISFACTORY |  | 10 | 1 | 346 | SATISFACTORY |
| 10 | 0 | 562 | SATISFACTORY |  | 10 | 1 | 692 | SATISFACTORY |
| 10 | 1 | 1778 | SATISFACTORY |  | 10 | 0 | 427 | SATISFACTORY |
| 10 | 0 | 5012 | SATISFACTORY |  | 10 | 0 | 3162 | SATISFACTORY |
| 10 | 0 | 178 | SATISFACTORY |  | 10 | 1 | 240 | SATISFACTORY |
| 10 | 0 | 56 | SATISFACTORY |  | 10 | 0 | 2371 | SATISFACTORY |
| 10 | 0 | 3162 | SATISFACTORY |  | 10 | 1 | 2154 | SATISFACTORY |
| 10 | 2 | 1600 | SATISFACTORY |  | 10 | 0 | 316 | SATISFACTORY |
| 10 | 0 | 562 | SATISFACTORY |  | 10 | 0 | 3162 | SATISFACTORY |
| 10 | 0 | 10000 | SATISFACTORY |  | 10 | 1 | 681 | SATISFACTORY |
| 10 | 1 | 1000 | SATISFACTORY |  | 10 | 0 | 562 | SATISFACTORY |
| 10 | 0 | 422 | SATISFACTORY |  | 10 | 0 | 1202 | SATISFACTORY |
| 10 | 0 | 480 | Inconclusive |  | 10 | 0 | 562 | SATISFACTORY |
| 10 | 1 | 1000 | SATISFACTORY |  | 10 | 0 | 351 | SATISFACTORY |
| 10 | 0 | 422 | SATISFACTORY |  | 10 | 0 | 3162 | SATISFACTORY |
| 10 | 0 | 1468 | SATISFACTORY |  | 10 | 0 | 1778 | SATISFACTORY |
| 10 | 0 | 2154 | SATISFACTORY |  | 10 | 1 | 681 | SATISFACTORY |
| 10 | 0 | 1468 | SATISFACTORY |  | 10 | 0 | 1000 | SATISFACTORY |
| 10 | 0 | 203 | SATISFACTORY |  | 10 | 0 | 1000 | SATISFACTORY |
| 10 | 0 | 147 | SATISFACTORY |  | 10 | 0 | 351 | SATISFACTORY |
| 10 | 0 | 5623 | SATISFACTORY |  | 10 | 0 | 100 | SATISFACTORY |
| 10 | 1 | 250 | SATISFACTORY |  | 10 | 1 | 2154 | SATISFACTORY |
| 10 | 0 | 1000 | SATISFACTORY |  | 10 | 0 | 178 | SATISFACTORY |
| 10 | 0 | 100 | SATISFACTORY |  | 10 | 0 | 1000 | SATISFACTORY |
| 10 | 1 | 234 | SATISFACTORY |  | 10 | 0 | 316 | SATISFACTORY |
| 10 | 0 | 2371 | SATISFACTORY |  | 10 | 0 | 1778 | SATISFACTORY |
| 10 | 0 | 100 | SATISFACTORY |  | 10 | 0 | 2089 | SATISFACTORY |
| 10 | 0 | 316 | SATISFACTORY |  | 10 | 0 | 3162 | SATISFACTORY |
| 10 | 1 | 178 | SATISFACTORY |  | 10 | 1 | 234 | SATISFACTORY |
| 10 | 0 | 6813 | SATISFACTORY |  | 10 | 0 | 3511 | SATISFACTORY |
| 10 | 0 | 2581 | SATISFACTORY |  | 10 | 0 | 1778 | SATISFACTORY |
| 10 | 0 | 316 | SATISFACTORY |  | 10 | 0 | 237 | SATISFACTORY |
| 10 | 0 | 481 | SATISFACTORY |  | 10 | 0 | 3162 | SATISFACTORY |
| 10 | 0 | 304 | SATISFACTORY |  | 10 | 1 | 1000 | SATISFACTORY |
| 10 | 0 | 2154 | SATISFACTORY |  | 10 | 1 | 59 | SATISFACTORY |
| 10 | 0 | 316 | SATISFACTORY |  | 10 | 0 | 631 | SATISFACTORY |
| 10 | 1 | 427 | SATISFACTORY |  | 10 | 0 | 562 | SATISFACTORY |
| 10 | 2 | 25 | SATISFACTORY |  | 10 | 0 | 1000 | SATISFACTORY |
| 10 | 1 | 18 | SATISFACTORY |  | 10 | 0 | 1778 | SATISFACTORY |
| 10 | 0 | 100 | SATISFACTORY |  | 10 | 0 | 3511 | SATISFACTORY |
| 10 | 1 | 2154 | SATISFACTORY |  | 10 | 0 | 1778 | SATISFACTORY |
| 10 | 0 | 1000 | No Test |  | 10 | 1 | 178 | SATISFACTORY |
| 10 | 0 | 316 | SATISFACTORY |  | 10 | 1 | 1000 | SATISFACTORY |
| 10 | 0 | 1468 | SATISFACTORY |  | 10 | 0 | 562 | SATISFACTORY |
| 10 | 0 | 46 | SATISFACTORY |  | 10 | 0 | 316 | SATISFACTORY |
| 10 | 0 | 562 | SATISFACTORY |  | 10 | 0 | 562 | SATISFACTORY |
| 10 | 0 | 624 | SATISFACTORY |  | 10 | 0 | 1000 | SATISFACTORY |
| 10 | 0 | 681 | SATISFACTORY |  | 10 | 0 | 2093 | SATISFACTORY |
| 10 | 0 | 75 | SATISFACTORY |  | 10 | 1 | 2371 | SATISFACTORY |
| 10 | 0 | 562 | SATISFACTORY |  | 10 | 0 | 1000 | SATISFACTORY |
| 10 | 1 | 215 | SATISFACTORY |  | 10 | 0 | 1000 | SATISFACTORY |
| 10 | 1 | 676 | SATISFACTORY |  | 10 | 0 | 3162 | SATISFACTORY |
| 10 | 1 | 481 | SATISFACTORY |  | 10 | 0 | 3162 | SATISFACTORY |
| 10 | 0 | 2371 | SATISFACTORY |  | 10 | 0 | 316 | SATISFACTORY |
| 10 | 0 | 1468 | SATISFACTORY |  | 10 | 0 | 2276 | SATISFACTORY |
| 10 | 1 | 178 | SATISFACTORY |  | 10 | 1 | 4266 | SATISFACTORY |
| 10 | 0 | 4169 | SATISFACTORY |  | 10 | 0 | 427 | SATISFACTORY |
| 10 | 0 | 178 | SATISFACTORY |  | 10 | 0 | 2138 | SATISFACTORY |
| 10 | 0 | 5495 | SATISFACTORY |  | 10 | 1 | 1000 | SATISFACTORY |
| 10 | 0 | 4266 | SATISFACTORY |  | 10 | 1 | 481 | SATISFACTORY |
| 10 | 0 | 681 | SATISFACTORY |  | 10 | 0 | 215 | SATISFACTORY |
| 10 | 0 | 316 | SATISFACTORY |  | 10 | 1 | 1000 | SATISFACTORY |
| 10 | 1 | 100 | SATISFACTORY |  | 10 | 0 | 427 | SATISFACTORY |
| 10 | 0 | 479 | SATISFACTORY |  | 10 | 1 | 178 | SATISFACTORY |
| 10 | 1 | 351 | SATISFACTORY |  | 10 | 0 | 3162 | SATISFACTORY |
| 10 | 0 | 316 | SATISFACTORY |  | 10 | 0 | 692 | SATISFACTORY |
| 10 | 1 | 4217 | SATISFACTORY |  | 10 | 0 | 1501 | SATISFACTORY |
| 10 | 0 | 1000 | SATISFACTORY |  | 10 | 1 | 228 | SATISFACTORY |
| 10 | 0 | 56 | SATISFACTORY |  | 10 | 2 | 1995 | SATISFACTORY |
| 10 | 0 | 464 | SATISFACTORY |  | 10 | 0 | 1468 | SATISFACTORY |
| 10 | 0 | 178 | SATISFACTORY |  | 10 | 2 | 182 | SATISFACTORY |
| 10 | 1 | 491 | SATISFACTORY |  | 10 | 0 | 351 | SATISFACTORY |
| 10 | 2 | 3715 | SATISFACTORY |  | 10 | 0 | 1778 | SATISFACTORY |
| 10 | 0 | 2344 | SATISFACTORY |  | 10 | 0 | 3511 | SATISFACTORY |
| 10 | 3 | 2 | NO TEST |  | 10 | 1 | 422 | SATISFACTORY |
| 10 | 0 | 1000 | SATISFACTORY |  | 10 | 0 | 588 | SATISFACTORY |
| 10 | 0 | 1000 | SATISFACTORY |  | 10 | 0 | 2089 | SATISFACTORY |
| 10 | 0 | 178 | SATISFACTORY |  | 10 | 0 | 200 | SATISFACTORY |
| 10 | 0 | 179 | SATISFACTORY |  | 10 | 1 | 1000 | SATISFACTORY |
| 10 | 2 | 1000 | SATISFACTORY |  | 10 | 0 | 1000 | SATISFACTORY |
| 10 | 0 | 46 | SATISFACTORY |  | 10 | 0 | 681 | SATISFACTORY |
| 10 | 1 | 422 | SATISFACTORY |  | 10 | 0 | 237 | SATISFACTORY |
| 10 | 1 | 100 | SATISFACTORY |  | 10 | 0 | 676 | SATISFACTORY |
| 10 | 0 | 200 | SATISFACTORY |  | 10 | 0 | 3511 | SATISFACTORY |
| 10 | 0 | 1000 | SATISFACTORY |  | 10 | 0 | 562 | SATISFACTORY |
| 10 | 0 | 3162 | SATISFACTORY |  | 10 | 1 | 178 | SATISFACTORY |
| 10 | 0 | 178 | SATISFACTORY |  | 10 | 0 | 351 | SATISFACTORY |
| 10 | 1 | 346 | SATISFACTORY |  | 10 | 0 | 3162 | SATISFACTORY |
| 10 | 0 | 215 | SATISFACTORY |  | 10 | 0 | 3511 | SATISFACTORY |
| 10 | 1 | 1778 | SATISFACTORY |  | 10 | 0 | 200 | SATISFACTORY |
| 10 | 0 | 316 | SATISFACTORY |  | 10 | 0 | 178 | SATISFACTORY |
| 10 | 1 | 32 | SATISFACTORY |  | 10 | 1 | 481 | SATISFACTORY |
| 10 | 0 | 178 | SATISFACTORY |  | 10 | 1 | 234 | SATISFACTORY |
| 10 | 0 | 2089 | SATISFACTORY |  | 10 | 0 | 1778 | SATISFACTORY |
| 10 | 0 | 562 | SATISFACTORY |  | 10 | 0 | 562 | SATISFACTORY |
| 10 | 0 | 316 | SATISFACTORY |  | 10 | 0 | 351 | SATISFACTORY |
| 10 | 0 | 215 | SATISFACTORY |  | 10 | 0 | 562 | SATISFACTORY |
| 10 | 0 | 2371 | SATISFACTORY |  | 10 | 0 | 5623 | SATISFACTORY |
| 10 | 0 | 10000 | SATISFACTORY |  | 10 | 1 | 228 | SATISFACTORY |
| 10 | 0 | 501 | Inconclusive |  | 10 | 0 | 3162 | SATISFACTORY |
| 10 | 0 | 464 | SATISFACTORY |  | 10 | 0 | 624 | SATISFACTORY |
| 10 | 0 | 562 | SATISFACTORY |  | 10 | 0 | 427 | SATISFACTORY |
| 10 | 1 | 351 | SATISFACTORY |  | 10 | 0 | 3511 | SATISFACTORY |
| 10 | 0 | 178 | SATISFACTORY |  | 10 | 0 | 3162 | SATISFACTORY |
| 10 | 2 | 23 | SATISFACTORY |  | 10 | 0 | 3311 | SATISFACTORY |
| 10 | 0 | 2081 | SATISFACTORY |  | 10 | 0 | 2512 | Inconclusive |
| 10 | 0 | 427 | SATISFACTORY |  | 10 | 0 | 2064 | SATISFACTORY |
| 10 | 1 | 200 | SATISFACTORY |  | 10 | 0 | 50 | SATISFACTORY |
| 10 | 0 | 316 | SATISFACTORY |  | 10 | 1 | 178 | SATISFACTORY |
| 10 | 1 | 1468 | SATISFACTORY |  | 10 | 1 | 631 | SATISFACTORY |
| 10 | 0 | 178 | SATISFACTORY |  | 10 | 0 | 3511 | SATISFACTORY |
| 10 | 1 | 200 | SATISFACTORY |  | 10 | 0 | 3311 | SATISFACTORY |
| 10 | 1 | 351 | SATISFACTORY |  | 10 | 0 | 3511 | SATISFACTORY |
| 10 | 1 | 215 | SATISFACTORY |  | 10 | 1 | 215 | SATISFACTORY |
| 10 | 1 | 179 | SATISFACTORY |  | 10 | 0 | 347 | SATISFACTORY |
| 10 | 1 | 159 | SATISFACTORY |  | 10 | 1 | 422 | SATISFACTORY |
| 10 | 1 | 178 | SATISFACTORY |  | 10 | 0 | 562 | SATISFACTORY |
| 10 | 1 | 100 | SATISFACTORY |  | 10 | 0 | 1778 | SATISFACTORY |
| 10 | 0 | 200 | SATISFACTORY |  | 10 | 1 | 215 | SATISFACTORY |
| 10 | 0 | 178 | SATISFACTORY |  | 10 | 0 | 2198 | SATISFACTORY |
| 10 | 0 | 316 | SATISFACTORY |  | 10 | 1 | 145 | SATISFACTORY |
| 10 | 0 | 32 | SATISFACTORY |  | 10 | 0 | 550 | SATISFACTORY |
| 10 | 0 | 3162 | SATISFACTORY |  | 10 | 0 | 562 | SATISFACTORY |
| 10 | 1 | 100 | SATISFACTORY |  | 10 | 0 | 3162 | SATISFACTORY |
| 10 | 1 | 151 | SATISFACTORY |  | 10 | 0 | 481 | SATISFACTORY |
| 10 | 0 | 2569 | SATISFACTORY |  | 10 | 0 | 1000 | NO TEST |
| 10 | 0 | 422 | SATISFACTORY |  | 10 | 0 | 794 | SATISFACTORY |
| 10 | 1 | 35 | SATISFACTORY |  | 10 | 0 | 1468 | SATISFACTORY |
| 10 | 0 | 794 | SATISFACTORY |  | 10 | 0 | 3020 | SATISFACTORY |
| 10 | 0 | 2371 | SATISFACTORY |  | 10 | 0 | 351 | SATISFACTORY |
| 10 | 0 | 4169 | SATISFACTORY |  | 10 | 0 | 3162 | SATISFACTORY |
| 10 | 1 | 422 | SATISFACTORY |  | 10 | 0 | 178 | SATISFACTORY |
| 10 | 0 | 1000 | SATISFACTORY |  | 10 | 0 | 3162 | SATISFACTORY |
| 10 | 0 | 316 | SATISFACTORY |  | 10 | 0 | 3162 | SATISFACTORY |
| 10 | 0 | 1778 | SATISFACTORY |  | 10 | 0 | 3162 | SATISFACTORY |
| 10 | 0 | 1000 | SATISFACTORY |  | 10 | 1 | 1000 | SATISFACTORY |
| 10 | 0 | 48 | SATISFACTORY |  | 10 | 0 | 3511 | SATISFACTORY |
| 10 | 0 | 316 | SATISFACTORY |  | 10 | 1 | 59 | SATISFACTORY |
| 10 | 0 | 4786 | SATISFACTORY |  | 10 | 0 | 1259 | SATISFACTORY |
| 10 | 0 | 316 | SATISFACTORY |  | 10 | 0 | 3162 | SATISFACTORY |
| 10 | 0 | 316 | SATISFACTORY |  | 10 | 0 | 3162 | SATISFACTORY |
| 10 | 0 | 18 | SATISFACTORY |  | 10 | 0 | 316 | SATISFACTORY |
| 10 | 0 | 562 | SATISFACTORY |  | 10 | 0 | 422 | SATISFACTORY |
| 10 | 0 | 178 | SATISFACTORY |  | 10 | 0 | 316 | SATISFACTORY |
| 10 | 0 | 316 | SATISFACTORY |  | 10 | 1 | 501 | SATISFACTORY |
| 10 | 0 | 562 | SATISFACTORY |  | 10 | 0 | 1778 | SATISFACTORY |
| 10 | 1 | 351 | SATISFACTORY |  | 10 | 0 | 3162 | SATISFACTORY |
| 10 | 0 | 3162 | SATISFACTORY |  | 10 | 1 | 481 | SATISFACTORY |
| 10 | 0 | 209 | SATISFACTORY |  | 10 | 2 | 258 | SATISFACTORY |
| 10 | 1 | 147 | SATISFACTORY |  | 10 | 0 | 427 | SATISFACTORY |
| 10 | 0 | 3162 | SATISFACTORY |  | 10 | 0 | 178 | SATISFACTORY |
| 10 | 0 | 316 | SATISFACTORY |  | 10 | 1 | 562 | SATISFACTORY |
| 10 | 0 | 3162 | SATISFACTORY |  | 10 | 0 | 316 | SATISFACTORY |
| 10 | 0 | 10000 | SATISFACTORY |  | 10 | 0 | 3162 | SATISFACTORY |
| 10 | 0 | 10000 | SATISFACTORY |  | 10 | 0 | 4806 | SATISFACTORY |
| 10 | 0 | 398 | SATISFACTORY |  | 10 | 0 | 3162 | SATISFACTORY |
| 10 | 0 | 4169 | SATISFACTORY |  | 10 | 0 | 234 | SATISFACTORY |
| 10 | 0 | 2081 | SATISFACTORY |  | 10 | 0 | 316 | SATISFACTORY |
| 10 | 0 | 681 | SATISFACTORY |  | 10 | 0 | 178 | SATISFACTORY |
| 10 | 1 | 18 | SATISFACTORY |  | 10 | 0 | 3162 | SATISFACTORY |
| 10 | 1 | 1000 | SATISFACTORY |  | 10 | 0 | 316 | SATISFACTORY |
| 10 | 0 | 1995 | SATISFACTORY |  | 10 | 1 | 178 | SATISFACTORY |
| 10 | 0 | 7943 | SATISFACTORY |  | 10 | 0 | 3162 | SATISFACTORY |
| 10 | 2 | 25 | SATISFACTORY |  | 10 | 0 | 562 | SATISFACTORY |
| 10 | 0 | 501 | SATISFACTORY |  | 10 | 0 | 1468 | SATISFACTORY |
| 10 | 0 | 10 | SATISFACTORY |  | 10 | 0 | 1259 | SATISFACTORY |
| 10 | 1 | 215 | SATISFACTORY |  | 10 | 0 | 237 | SATISFACTORY |
| 10 | 1 | 1000 | SATISFACTORY |  | 10 | 1 | 178 | SATISFACTORY |
| 10 | 0 | 7244 | SATISFACTORY |  | 10 | 0 | >10000 | NO TEST |
| 10 | 0 | 562 | SATISFACTORY |  | 10 | 0 | 1738 | SATISFACTORY |
| 10 | 0 | 178 | SATISFACTORY |  | 10 | 1 | 1000 | SATISFACTORY |
| 10 | 0 | 100 | SATISFACTORY |  | 10 | 0 | 316 | SATISFACTORY |
| 10 | 0 | 316 | SATISFACTORY |  | 10 | 0 | 1778 | SATISFACTORY |
| 10 | 1 | 422 | SATISFACTORY |  | 10 | 0 | 1000 | SATISFACTORY |
| 10 | 0 | 316 | SATISFACTORY |  | 10 | 0 | 3162 | SATISFACTORY |
| 10 | 2 | 1000 | SATISFACTORY |  | 10 | 0 | 316 | SATISFACTORY |
| 10 | 0 | 351 | SATISFACTORY |  | 10 | 0 | 474 | SATISFACTORY |
| 10 | 2 | 1600 | SATISFACTORY |  | 10 | 0 | 6310 | SATISFACTORY |
| 10 | 0 | 228 | SATISFACTORY |  | 10 | 0 | 1000 | SATISFACTORY |
| 10 | 0 | 481 | SATISFACTORY |  | 10 | 0 | 316 | SATISFACTORY |
| 10 | 1 | 351 | SATISFACTORY |  | 10 | 1 | 422 | SATISFACTORY |
| 10 | 1 | 2154 | SATISFACTORY |  | 10 | 0 | 1585 | SATISFACTORY |
| 10 | 0 | 562 | SATISFACTORY |  | 10 | 0 | 1259 | SATISFACTORY |
| 10 | 0 | 3162 | SATISFACTORY |  | 10 | 0 | 1585 | SATISFACTORY |
| 10 | 0 | 422 | SATISFACTORY |  | 10 | 0 | 562 | SATISFACTORY |
| 10 | 0 | 316 | SATISFACTORY |  | 10 | 0 | 316 | SATISFACTORY |
| 10 | 1 | 692 | SATISFACTORY |  | 10 | 0 | 100 | SATISFACTORY |
| 10 | 0 | 3162 | SATISFACTORY |  | 10 | 0 | 3162 | SATISFACTORY |
| 10 | 0 | 351 | SATISFACTORY |  | 10 | 0 | 1442 | SATISFACTORY |
| 10 | 2 | 3715 | SATISFACTORY |  | 10 | 1 | 145 | SATISFACTORY |
| 10 | 0 | 2154 | SATISFACTORY |  | 10 | 0 | 3162 | SATISFACTORY |
| 10 | 0 | 316 | SATISFACTORY |  | 10 | 0 | 178 | SATISFACTORY |
| 10 | 0 | 2154 | SATISFACTORY |  | 10 | 0 | 1259 | SATISFACTORY |
| 10 | 0 | 562 | SATISFACTORY |  | 10 | 0 | 3162 | SATISFACTORY |
| 10 | 1 | 6310 | SATISFACTORY |  | 10 | 0 | 1778 | SATISFACTORY |
| 10 | 1 | 422 | SATISFACTORY |  | 10 | 0 | 329 | SATISFACTORY |
| 10 | 0 | 3162 | SATISFACTORY |  | 10 | 0 | 50 | SATISFACTORY |
| 10 | 0 | 624 | SATISFACTORY |  | 10 | 1 | 59 | SATISFACTORY |
| 10 | 1 | 178 | SATISFACTORY |  | 10 | 2 | 3162 | SATISFACTORY |
| 10 | 0 | 100 | SATISFACTORY |  | 10 | 1 | 59 | SATISFACTORY |
| 10 | 1 | 100 | SATISFACTORY |  | 10 | 0 | 3162 | SATISFACTORY |
| 10 | 1 | 42 | SATISFACTORY |  | 10 | 1 | 316 | SATISFACTORY |
| 10 | 0 | 562 | SATISFACTORY |  | 10 | 0 | 794 | SATISFACTORY |
| 10 | 0 | 5012 | SATISFACTORY |  | 10 | 0 | 1000 | SATISFACTORY |
| 10 | 0 | 6607 | SATISFACTORY |  | 10 | 1 | 178 | SATISFACTORY |
| 10 | 0 | 3162 | SATISFACTORY |  | 10 | 0 | 3162 | SATISFACTORY |
| 10 | 0 | 621 | SATISFACTORY |  | 10 | 0 | 3162 | SATISFACTORY |
| 10 | 0 | 562 | SATISFACTORY |  | 10 | 0 | 316 | SATISFACTORY |
| 10 | 1 | 422 | SATISFACTORY |  | 10 | 1 | 2154 | SATISFACTORY |
| 10 | 1 | 1479 | SATISFACTORY |  | 10 | 0 | 3162 | SATISFACTORY |
| 10 | 1 | 332 | SATISFACTORY |  | 10 | 0 | 1000 | SATISFACTORY |
| 10 | 0 | 316 | SATISFACTORY |  | 10 | 0 | 316 | SATISFACTORY |
| 10 | 1 | 422 | SATISFACTORY |  | 10 | 0 | 316 | SATISFACTORY |
| 10 | 0 | 2154 | SATISFACTORY |  | 10 | 0 | 588 | SATISFACTORY |
| 10 | 0 | 501 | SATISFACTORY |  | 10 | 1 | 1327 | SATISFACTORY |
| 10 | 0 | 3495 | SATISFACTORY |  | 10 | 1 | 427 | SATISFACTORY |
| 10 | 0 | 422 | SATISFACTORY |  | 10 | 0 | 2093 | SATISFACTORY |
| 10 | 0 | 562 | SATISFACTORY |  | 10 | 1 | 481 | SATISFACTORY |
| 10 | 0 | 6607 | SATISFACTORY |  | 10 | 0 | 3162 | SATISFACTORY |
| 10 | 1 | 178 | SATISFACTORY |  | 10 | 0 | 1000 | SATISFACTORY |
| 10 | 0 | 3162 | SATISFACTORY |  | 10 | 0 | 558 | SATISFACTORY |
| 10 | 0 | 3162 | SATISFACTORY |  | 10 | 0 | 215 | SATISFACTORY |
| 10 | 0 | 5012 | SATISFACTORY |  | 10 | 1 | 501 | SATISFACTORY |
| 10 | 1 | 351 | SATISFACTORY |  | 10 | 0 | 3162 | SATISFACTORY |
| 10 | 0 | 3162 | SATISFACTORY |  | 10 | 0 | 1000 | SATISFACTORY |
| 10 | 0 | 501 | SATISFACTORY |  | 10 | 0 | 562 | SATISFACTORY |
| 10 | 0 | 428 | SATISFACTORY |  | 10 | 0 | 562 | SATISFACTORY |
| 10 | 0 | 329 | SATISFACTORY |  | 10 | 1 | 59 | SATISFACTORY |
| 10 | 0 | 316 | SATISFACTORY |  | 10 | 0 | 3162 | SATISFACTORY |
| 10 | 1 | 351 | SATISFACTORY |  | 10 | 0 | 3162 | SATISFACTORY |
| 10 | 0 | 316 | SATISFACTORY |  | 10 | 1 | 1000 | SATISFACTORY |
| 10 | 0 | 464 | SATISFACTORY |  | 10 | 2 | 228 | SATISFACTORY |
| 10 | 0 | 100 | SATISFACTORY |  | 10 | 0 | >10000 | NO TEST |
| 10 | 0 | 316 | SATISFACTORY |  | 10 | 0 | 178 | SATISFACTORY |
| 10 | 0 | 316 | SATISFACTORY |  | 10 | 0 | 347 | SATISFACTORY |
| 10 | 1 | 351 | SATISFACTORY |  | 10 | 0 | 441 | SATISFACTORY |
| 10 | 1 | 422 | SATISFACTORY |  | 10 | 0 | 351 | SATISFACTORY |
| 10 | 0 | 3162 | SATISFACTORY |  | 10 | 1 | 422 | SATISFACTORY |
| 10 | 0 | 215 | SATISFACTORY |  | 10 | 0 | 4806 | SATISFACTORY |
| 10 | 0 | 178 | SATISFACTORY |  | 10 | 0 | 3162 | SATISFACTORY |
| 10 | 0 | 1000 | SATISFACTORY |  | 10 | 2 | 1995 | SATISFACTORY |
| 10 | 0 | 10000 | SATISFACTORY |  | 10 | 0 | 2093 | SATISFACTORY |
| 10 | 1 | 1778 | SATISFACTORY |  | 10 | 0 | 1000 | SATISFACTORY |
| 10 | 1 | 351 | SATISFACTORY |  | 10 | 0 | 3162 | SATISFACTORY |
| 10 | 0 | 1000 | SATISFACTORY |  | 10 | 0 | 464 | SATISFACTORY |
| 10 | 0 | 562 | SATISFACTORY |  | 10 | 1 | 228 | SATISFACTORY |
| 10 | 0 | 126 | SATISFACTORY |  | 10 | 0 | 3162 | SATISFACTORY |
| 10 | 0 | 501 | SATISFACTORY |  | 10 | 1 | 1000 | SATISFACTORY |
| 10 | 0 | 178 | SATISFACTORY |  | 10 | 0 | 3162 | SATISFACTORY |
| 10 | 0 | 562 | SATISFACTORY |  | 10 | 0 | 1000 | SATISFACTORY |
| 10 | 0 | 3162 | SATISFACTORY |  | 10 | 0 | 3162 | SATISFACTORY |
| 10 | 0 | 1468 | SATISFACTORY |  | 10 | 0 | 3162 | SATISFACTORY |
| 10 | 0 | 178 | SATISFACTORY |  | 10 | 0 | 1778 | SATISFACTORY |
| 10 | 1 | 351 | SATISFACTORY |  | 10 | 0 | 50 | SATISFACTORY |
| 10 | 0 | 316 | SATISFACTORY |  | 10 | 0 | 1000 | SATISFACTORY |
| 10 | 0 | 1000 | SATISFACTORY |  | 10 | 1 | 2089 | SATISFACTORY |
| 10 | 1 | 1778 | SATISFACTORY |  | 10 | 0 | 398 | SATISFACTORY |
| 10 | 0 | 316 | SATISFACTORY |  | 10 | 0 | 215 | SATISFACTORY |
| 10 | 1 | 215 | SATISFACTORY |  | 10 | 0 | 3162 | SATISFACTORY |
| 10 | 0 | 562 | SATISFACTORY |  | 10 | 0 | 2512 | SATISFACTORY |
| 10 | 0 | 1778 | SATISFACTORY |  | 10 | 2 | 316 | SATISFACTORY |
| 10 | 0 | 200 | SATISFACTORY |  | 10 | 0 | 1000 | SATISFACTORY |
| 10 | 0 | 10001 | NO TEST |  | 10 | 0 | 1778 | SATISFACTORY |
| 10 | 0 | 32 | SATISFACTORY |  | 10 | 0 | 3162 | SATISFACTORY |
| 10 | 0 | 562 | SATISFACTORY |  | 10 | 0 | 588 | SATISFACTORY |
| 10 | 0 | 3162 | SATISFACTORY |  | 10 | 0 | 200 | SATISFACTORY |
| 10 | 1 | 215 | SATISFACTORY |  | 10 | 0 | 2093 | SATISFACTORY |
| 10 | 0 | 316 | SATISFACTORY |  | 10 | 0 | 1000 | SATISFACTORY |
| 10 | 1 | 178 | SATISFACTORY |  | 10 | 0 | 215 | SATISFACTORY |
| 10 | 0 | 4786 | SATISFACTORY |  | 10 | 0 | 1000 | SATISFACTORY |
| 10 | 1 | 179 | SATISFACTORY |  | 10 | 0 | 3162 | SATISFACTORY |
| 10 | 2 | 1000 | SATISFACTORY |  | 10 | 0 | 316 | SATISFACTORY |
| 10 | 0 | 178 | SATISFACTORY |  | 10 | 0 | 63 | SATISFACTORY |
| 10 | 0 | 1778 | SATISFACTORY |  | 10 | 0 | 681 | SATISFACTORY |
| 10 | 0 | 422 | SATISFACTORY |  | 10 | 0 | 1738 | SATISFACTORY |
| 10 | 0 | 32 | SATISFACTORY |  | 10 | 0 | 1000 | SATISFACTORY |
| 10 | 0 | 2371 | Inconclusive |  | 10 | 1 | 1000 | SATISFACTORY |
| 10 | 0 | 2081 | SATISFACTORY |  | 10 | 0 | 562 | SATISFACTORY |
| 10 | 0 | 178 | SATISFACTORY |  | 10 | 0 | 1000 | SATISFACTORY |
| 10 | 1 | 42 | SATISFACTORY |  | 10 | 1 | 178 | SATISFACTORY |
| 10 | 1 | 681 | SATISFACTORY |  | 10 | 0 | 631 | SATISFACTORY |
| 10 | 0 | 10001 | NO TEST |  | 10 | 2 | 40 | SATISFACTORY |
| 10 | 0 | 316 | SATISFACTORY |  | 10 | 0 | 464 | SATISFACTORY |
| 10 | 0 | 237 | SATISFACTORY |  | 10 | 0 | 1468 | SATISFACTORY |
| 10 | 1 | 481 | SATISFACTORY |  | 10 | 1 | 1000 | SATISFACTORY |
| 10 | 0 | 3162 | SATISFACTORY |  | 10 | 0 | 215 | SATISFACTORY |
| 10 | 0 | 478 | SATISFACTORY |  | 10 | 0 | 562 | SATISFACTORY |
| 10 | 0 | 10000 | SATISFACTORY |  | 10 | 1 | 63 | SATISFACTORY |
| 10 | 0 | 1000 | NO TEST |  | 10 | 0 | 1778 | SATISFACTORY |
| 10 | 0 | 3162 | SATISFACTORY |  | 10 | 1 | 3325 | SATISFACTORY |
| 10 | 0 | 178 | SATISFACTORY |  | 10 | 0 | 316 | SATISFACTORY |
| 10 | 0 | 215 | SATISFACTORY |  | 10 | 0 | 1778 | NO TEST |
| 10 | 0 | 2154 | SATISFACTORY |  | 10 | 0 | 562 | SATISFACTORY |
| 10 | 0 | 178 | SATISFACTORY |  | 10 | 0 | 562 | SATISFACTORY |
| 10 | 0 | 316 | SATISFACTORY |  | 10 | 1 | 2089 | SATISFACTORY |
| 10 | 0 | 10 | SATISFACTORY |  | 10 | 0 | 1000 | SATISFACTORY |
| 10 | 0 | 6043 | SATISFACTORY |  | 10 | 0 | 3162 | SATISFACTORY |
| 10 | 1 | 100 | SATISFACTORY |  | 10 | 0 | 3162 | SATISFACTORY |
| 10 | 0 | 316 | SATISFACTORY |  | 10 | 0 | 316 | SATISFACTORY |
| 10 | 1 | 2540 | SATISFACTORY |  | 10 | 0 | 1000 | SATISFACTORY |
| 10 | 1 | 422 | SATISFACTORY |  | 10 | 0 | 316 | SATISFACTORY |
| 10 | 0 | 147 | SATISFACTORY |  | 10 | 0 | 316 | SATISFACTORY |
| 10 | 0 | 681 | SATISFACTORY |  | 10 | 0 | 3162 | SATISFACTORY |
| 10 | 0 | 2154 | SATISFACTORY |  | 10 | 0 | 588 | SATISFACTORY |
| 10 | 0 | 422 | SATISFACTORY |  | 10 | 0 | 316 | SATISFACTORY |
| 10 | 0 | 316 | SATISFACTORY |  | 10 | 1 | 35 | SATISFACTORY |
| 10 | 0 | 215 | SATISFACTORY |  | 10 | 1 | 955 | SATISFACTORY |
| 10 | 0 | 550 | SATISFACTORY |  | 10 | 2 | 7244 | SATISFACTORY |
| 10 | 1 | 147 | SATISFACTORY |  | 10 | 0 | 178 | SATISFACTORY |
| 10 | 0 | 6607 | SATISFACTORY |  | 10 | 0 | 3162 | SATISFACTORY |
| 10 | 0 | 562 | SATISFACTORY |  | 10 | 0 | 1000 | SATISFACTORY |
| 10 | 0 | 10 | SATISFACTORY |  | 10 | 0 | 3162 | SATISFACTORY |
| 10 | 2 | 63 | SATISFACTORY |  | 10 | 0 | 681 | SATISFACTORY |
| 10 | 0 | 316 | SATISFACTORY |  | 10 | 0 | 3162 | SATISFACTORY |
| 10 | 3 | 1607 | NO TEST |  | 10 | 1 | 422 | SATISFACTORY |
| 10 | 0 | 178 | SATISFACTORY |  | 10 | 0 | 3511 | SATISFACTORY |
| 10 | 0 | 464 | SATISFACTORY |  | 10 | 0 | 3162 | SATISFACTORY |
| 10 | 1 | 1778 | SATISFACTORY |  | 10 | 0 | 681 | SATISFACTORY |
| 10 | 0 | 3162 | SATISFACTORY |  | 10 | 1 | 1000 | SATISFACTORY |
| 10 | 0 | 562 | SATISFACTORY |  | 10 | 0 | 351 | SATISFACTORY |
| 10 | 0 | 179 | SATISFACTORY |  | 10 | 0 | 3511 | SATISFACTORY |
| 10 | 1 | 422 | SATISFACTORY |  | 10 | 0 | 699 | SATISFACTORY |
| 10 | 1 | 422 | SATISFACTORY |  | 10 | 1 | 59 | SATISFACTORY |
| 10 | 0 | 3162 | SATISFACTORY |  | 10 | 0 | 562 | SATISFACTORY |
| 10 | 0 | 3162 | SATISFACTORY |  | 10 | 0 | 3162 | SATISFACTORY |
| 10 | 0 | 1000 | Inconclusive |  | 10 | 0 | 209 | SATISFACTORY |
| 10 | 0 | 4786 | SATISFACTORY |  | 10 | 0 | 215 | SATISFACTORY |
| 10 | 1 | 178 | SATISFACTORY |  | 10 | 0 | 351 | SATISFACTORY |
| 10 | 0 | 178 | SATISFACTORY |  | 10 | 0 | 562 | SATISFACTORY |
| 10 | 0 | 3162 | SATISFACTORY |  | 10 | 0 | 422 | SATISFACTORY |
| 10 | 0 | 428 | SATISFACTORY |  | 10 | 0 | 562 | SATISFACTORY |
| 10 | 0 | 1738 | SATISFACTORY |  | 10 | 0 | 200 | SATISFACTORY |
| 10 | 0 | 2154 | SATISFACTORY |  | 10 | 1 | 700 | SATISFACTORY |
| 10 | 0 | 3162 | SATISFACTORY |  | 10 | 0 | 631 | SATISFACTORY |
| 10 | 0 | 178 | SATISFACTORY |  | 10 | 0 | 562 | SATISFACTORY |
| 10 | 0 | 3162 | SATISFACTORY |  | 10 | 0 | 588 | SATISFACTORY |
| 10 | 1 | 351 | SATISFACTORY |  | 10 | 0 | 603 | SATISFACTORY |
| 10 | 0 | 316 | SATISFACTORY |  | 10 | 0 | 316 | SATISFACTORY |
| 10 | 0 | 316 | SATISFACTORY |  | 10 | 1 | 228 | SATISFACTORY |
| 10 | 0 | 478 | SATISFACTORY |  | 10 | 1 | 240 | SATISFACTORY |
| 10 | 1 | 3981 | SATISFACTORY |  | 10 | 0 | 316 | SATISFACTORY |
| 10 | 0 | 1778 | SATISFACTORY |  | 10 | 1 | 1000 | SATISFACTORY |
| 10 | 1 | 351 | SATISFACTORY |  | 10 | 0 | 2154 | SATISFACTORY |
| 10 | 0 | 178 | SATISFACTORY |  | 10 | 0 | 1442 | SATISFACTORY |
| 10 | 0 | 215 | SATISFACTORY |  | 10 | 0 | 1778 | SATISFACTORY |
| 10 | 0 | 2081 | SATISFACTORY |  | 10 | 0 | 3162 | SATISFACTORY |
| 10 | 1 | 481 | SATISFACTORY |  | 10 | 0 | 1585 | SATISFACTORY |
| 10 | 1 | 18 | SATISFACTORY |  | 10 | 1 | 422 | SATISFACTORY |
| 10 | 0 | 316 | SATISFACTORY |  | 10 | 1 | 422 | SATISFACTORY |
| 10 | 0 | 178 | SATISFACTORY |  | 10 | 0 | 1000 | SATISFACTORY |
| 10 | 1 | 32 | SATISFACTORY |  | 10 | 0 | 3162 | SATISFACTORY |
| 10 | 1 | 351 | SATISFACTORY |  | 10 | 1 | 179 | SATISFACTORY |
| 10 | 0 | 178 | SATISFACTORY |  | 10 | 0 | 1000 | SATISFACTORY |
| 10 | 0 | 10000 | SATISFACTORY |  | 10 | 0 | 2512 | SATISFACTORY |
| 10 | 1 | 208 | SATISFACTORY |  | 10 | 0 | 562 | SATISFACTORY |
| 10 | 1 | 631 | SATISFACTORY |  | 10 | 0 | 316 | SATISFACTORY |
| 10 | 1 | 1778 | No Test |  | 10 | 0 | 3162 | SATISFACTORY |
| 10 | 0 | 178 | SATISFACTORY |  | 10 | 0 | 3162 | SATISFACTORY |
| 10 | 0 | 316 | SATISFACTORY |  | 10 | 0 | 3162 | SATISFACTORY |
| 10 | 1 | 251 | SATISFACTORY |  | 10 | 0 | 178 | SATISFACTORY |
| 10 | 0 | 3162 | SATISFACTORY |  | 10 | 0 | 147 | SATISFACTORY |
| 10 | 1 | 100 | SATISFACTORY |  | 10 | 0 | 316 | SATISFACTORY |
| 10 | 0 | 316 | SATISFACTORY |  | 10 | 0 | 562 | SATISFACTORY |
| 10 | 0 | 316 | SATISFACTORY |  | 10 | 0 | 4217 | SATISFACTORY |
| 10 | 0 | 215 | SATISFACTORY |  | 10 | 0 | 2093 | SATISFACTORY |
| 10 | 0 | 562 | SATISFACTORY |  | 10 | 0 | 1995 | SATISFACTORY |
| 10 | 1 | 100 | SATISFACTORY |  | 10 | 0 | 422 | SATISFACTORY |
| 10 | 1 | 1000 | SATISFACTORY |  | 10 | 0 | 3162 | SATISFACTORY |
| 10 | 0 | 1000 | SATISFACTORY |  | 10 | 1 | 599 | SATISFACTORY |
| 10 | 0 | 32 | SATISFACTORY |  | 10 | 0 | 351 | SATISFACTORY |
| 10 | 0 | 1778 | SATISFACTORY |  | 10 | 0 | 676 | SATISFACTORY |
| 10 | 0 | 1778 | SATISFACTORY |  | 10 | 0 | 1995 | SATISFACTORY |
| 10 | 0 | 178 | SATISFACTORY |  | 10 | 1 | 2371 | SATISFACTORY |
| 10 | 1 | 422 | SATISFACTORY |  | 10 | 0 | 208 | SATISFACTORY |
| 10 | 0 | 1778 | SATISFACTORY |  | 10 | 0 | 1468 | SATISFACTORY |
| 10 | 0 | 1468 | SATISFACTORY |  | 10 | 0 | 3162 | SATISFACTORY |
| 10 | 1 | 32 | SATISFACTORY |  | 10 | 0 | 316 | SATISFACTORY |
| 10 | 1 | 631 | SATISFACTORY |  | 10 | 0 | 100 | SATISFACTORY |
| 10 | 0 | 2081 | SATISFACTORY |  | 10 | 0 | 316 | SATISFACTORY |
| 10 | 0 | 316 | SATISFACTORY |  | 10 | 0 | 562 | SATISFACTORY |
| 10 | 0 | 18 | SATISFACTORY |  | 10 | 0 | 1000 | SATISFACTORY |
| 10 | 0 | 178 | SATISFACTORY |  | 10 | 0 | 464 | SATISFACTORY |
| 10 | 1 | 151 | SATISFACTORY |  | 10 | 1 | 1000 | SATISFACTORY |
| 10 | 1 | 351 | SATISFACTORY |  | 10 | 1 | 178 | SATISFACTORY |
| 10 | 0 | 179 | SATISFACTORY |  | 10 | 1 | 1000 | SATISFACTORY |
| 10 | 0 | 562 | SATISFACTORY |  | 10 | 0 | 215 | SATISFACTORY |
| 10 | 2 | 1000 | SATISFACTORY |  | 10 | 0 | 588 | SATISFACTORY |
| 10 | 0 | 562 | SATISFACTORY |  | 10 | 0 | 215 | SATISFACTORY |
| 10 | 0 | 351 | SATISFACTORY |  | 10 | 1 | 228 | SATISFACTORY |
| 10 | 1 | 422 | SATISFACTORY |  | 10 | 0 | 215 | SATISFACTORY |
| 10 | 0 | 316 | SATISFACTORY |  | 10 | 0 | 1445 | SATISFACTORY |
| 10 | 0 | 481 | SATISFACTORY |  | 10 | 0 | 3162 | SATISFACTORY |
| 10 | 1 | 481 | SATISFACTORY |  | 10 | 0 | 178 | SATISFACTORY |
| 10 | 1 | 468 | SATISFACTORY |  | 10 | 1 | 1000 | SATISFACTORY |
| 10 | 1 | 1000 | SATISFACTORY |  | 10 | 1 | 215 | SATISFACTORY |
| 10 | 1 | 126 | SATISFACTORY |  | 10 | 0 | 178 | SATISFACTORY |
| 10 | 0 | 1000 | SATISFACTORY |  | 10 | 1 | 422 | SATISFACTORY |
| 10 | 0 | 32 | SATISFACTORY |  | 10 | 0 | 3162 | SATISFACTORY |
| 10 | 0 | 350 | SATISFACTORY |  | 10 | 0 | 3162 | SATISFACTORY |
| 10 | 0 | 481 | SATISFACTORY |  | 10 | 1 | 1000 | SATISFACTORY |
| 10 | 0 | 464 | SATISFACTORY |  | 10 | 0 | 1259 | SATISFACTORY |
| 10 | 1 | 2154 | SATISFACTORY |  | 10 | 2 | 182 | SATISFACTORY |
| 10 | 0 | 2512 | SATISFACTORY |  | 10 | 0 | 215 | SATISFACTORY |
| 10 | 0 | 215 | SATISFACTORY |  | 10 | 0 | 316 | SATISFACTORY |
| 10 | 0 | 3162 | SATISFACTORY |  | 10 | 0 | 3162 | SATISFACTORY |
| 10 | 0 | 6607 | SATISFACTORY |  | 10 | 0 | 1000 | SATISFACTORY |
| 10 | 0 | 178 | SATISFACTORY |  | 10 | 1 | 1000 | SATISFACTORY |
| 10 | 0 | 237 | SATISFACTORY |  | 10 | 0 | 794 | SATISFACTORY |
| 10 | 1 | 422 | SATISFACTORY |  | 10 | 0 | 3162 | SATISFACTORY |
| 10 | 0 | 316 | SATISFACTORY |  | 10 | 1 | 178 | SATISFACTORY |
| 10 | 0 | 10001 | NO TEST |  | 10 | 0 | 681 | SATISFACTORY |
| 10 | 1 | 2154 | SATISFACTORY |  | 10 | 0 | 562 | SATISFACTORY |
| 10 | 1 | 1000 | SATISFACTORY |  | 10 | 1 | 562 | SATISFACTORY |
| 10 | 0 | 3162 | SATISFACTORY |  | 10 | 0 | 3162 | SATISFACTORY |
| 10 | 0 | 4266 | SATISFACTORY |  | 10 | 0 | 214 | SATISFACTORY |
| 10 | 0 | 10001 | NO TEST |  | 10 | 0 | 3162 | SATISFACTORY |
| 10 | 0 | 316 | SATISFACTORY |  | 10 | 0 | 2154 | SATISFACTORY |
| 10 | 0 | 316 | SATISFACTORY |  | 10 | 0 | 2371 | SATISFACTORY |
| 10 | 0 | 178 | SATISFACTORY |  | 10 | 0 | 464 | SATISFACTORY |
| 10 | 1 | 1778 | SATISFACTORY |  | 10 | 0 | 1778 | SATISFACTORY |
| 10 | 1 | 1479 | SATISFACTORY |  | 10 | 0 | 316 | SATISFACTORY |
| 10 | 1 | 491 | SATISFACTORY |  | 10 | 0 | 1096 | SATISFACTORY |
| 10 | 1 | 100 | SATISFACTORY |  | 10 | 0 | 316 | SATISFACTORY |
| 10 | 0 | 10000 | SATISFACTORY |  | 10 | 0 | 3162 | SATISFACTORY |
| 10 | 0 | 3162 | SATISFACTORY |  | 10 | 0 | 464 | SATISFACTORY |
| 10 | 0 | 6607 | SATISFACTORY |  | 10 | 0 | 316 | SATISFACTORY |
| 10 | 1 | 491 | SATISFACTORY |  | 10 | 0 | 3162 | SATISFACTORY |
| 10 | 1 | 1468 | SATISFACTORY |  | 10 | 1 | 178 | SATISFACTORY |
| 10 | 0 | 1468 | SATISFACTORY |  | 10 | 0 | 351 | SATISFACTORY |
| 10 | 0 | 2512 | SATISFACTORY |  | 10 | 1 | 32 | SATISFACTORY |
| 10 | 0 | 178 | SATISFACTORY |  | 10 | 0 | 178 | SATISFACTORY |
| 10 | 1 | 42 | SATISFACTORY |  | 10 | 1 | 1000 | SATISFACTORY |
| 10 | 0 | 398 | SATISFACTORY |  | 10 | 0 | 178 | SATISFACTORY |
| 10 | 0 | 46 | SATISFACTORY |  | 10 | 0 | 562 | SATISFACTORY |
| 10 | 0 | 3162 | SATISFACTORY |  | 10 | 0 | 316 | SATISFACTORY |
| 10 | 0 | 316 | SATISFACTORY |  | 10 | 0 | 351 | SATISFACTORY |
| 10 | 0 | 1778 | SATISFACTORY |  | 10 | 1 | 178 | SATISFACTORY |
| 10 | 0 | 316 | SATISFACTORY |  | 10 | 0 | 3162 | SATISFACTORY |
| 10 | 0 | 316 | SATISFACTORY |  | 10 | 0 | 178 | SATISFACTORY |
| 10 | 0 | 422 | SATISFACTORY |  | 10 | 1 | 1000 | SATISFACTORY |
| 10 | 1 | 35 | SATISFACTORY |  | 10 | 0 | 1468 | SATISFACTORY |
| 10 | 1 | 346 | SATISFACTORY |  | 10 | 0 | 2093 | SATISFACTORY |
| 10 | 0 | 3162 | SATISFACTORY |  | 10 | 0 | 215 | SATISFACTORY |
| 10 | 0 | 316 | SATISFACTORY |  | 10 | 0 | 1259 | SATISFACTORY |
| 10 | 0 | 178 | SATISFACTORY |  | 10 | 1 | 178 | SATISFACTORY |
| 10 | 0 | 329 | SATISFACTORY |  | 10 | 0 | 2371 | SATISFACTORY |
| 10 | 0 | 215 | SATISFACTORY |  | 10 | 0 | 316 | SATISFACTORY |
| 10 | 0 | 316 | SATISFACTORY |  | 10 | 0 | 3511 | SATISFACTORY |
| 10 | 1 | 422 | SATISFACTORY |  | 10 | 1 | 955 | SATISFACTORY |
| 10 | 0 | 3162 | SATISFACTORY |  | 10 | 0 | 562 | SATISFACTORY |
| 10 | 0 | 3162 | SATISFACTORY |  | 10 | 0 | 398 | SATISFACTORY |
| 10 | 0 | 240 | SATISFACTORY |  | 10 | 0 | 1778 | SATISFACTORY |
| 10 | 0 | 178 | SATISFACTORY |  | 10 | 1 | 1000 | SATISFACTORY |
| 10 | 0 | 240 | SATISFACTORY |  | 10 | 0 | 316 | SATISFACTORY |
| 10 | 1 | 422 | SATISFACTORY |  | 10 | 0 | 351 | SATISFACTORY |
| 10 | 1 | 468 | SATISFACTORY |  | 10 | 1 | 145 | SATISFACTORY |
| 10 | 0 | 100 | SATISFACTORY |  | 10 | 0 | 1000 | SATISFACTORY |
| 10 | 0 | 10000 | SATISFACTORY |  | 10 | 0 | 42 | SATISFACTORY |
| 10 | 0 | 100 | SATISFACTORY |  | 10 | 0 | 1000 | SATISFACTORY |
| 10 | 0 | 316 | SATISFACTORY |  | 10 | 0 | 316 | SATISFACTORY |
| 10 | 0 | 1468 | SATISFACTORY |  | 10 | 0 | 3162 | SATISFACTORY |
| 10 | 0 | 10000 | SATISFACTORY |  | 10 | 0 | 170 | SATISFACTORY |
| 10 | 0 | 237 | SATISFACTORY |  | 10 | 0 | 3162 | SATISFACTORY |
| 10 | 0 | 178 | SATISFACTORY |  | 10 | 0 | 1778 | SATISFACTORY |
| 10 | 0 | 316 | SATISFACTORY |  | 10 | 0 | 1000 | SATISFACTORY |
| 10 | 0 | 178 | SATISFACTORY |  | 10 | 1 | 2371 | SATISFACTORY |
| 10 | 0 | 562 | SATISFACTORY |  | 10 | 0 | 234 | SATISFACTORY |
| 10 | 1 | 351 | SATISFACTORY |  | 10 | 0 | 1000 | SATISFACTORY |
| 10 | 0 | 562 | SATISFACTORY |  | 10 | 1 | 422 | SATISFACTORY |
| 10 | 1 | 18 | SATISFACTORY |  | 10 | 0 | 2276 | SATISFACTORY |
| 10 | 0 | 316 | SATISFACTORY |  | 10 | 1 | 4074 | SATISFACTORY |
| 10 | 1 | 178 | SATISFACTORY |  | 10 | 0 | 2512 | SATISFACTORY |
| 10 | 1 | 1000 | SATISFACTORY |  | 10 | 1 | 234 | SATISFACTORY |
| 10 | 0 | 1655 | SATISFACTORY |  | 10 | 1 | 13 | SATISFACTORY |
| 10 | 0 | 32 | SATISFACTORY |  | 10 | 0 | 1608 | SATISFACTORY |
| 10 | 0 | 589 | SATISFACTORY |  | 10 | 1 | 1000 | SATISFACTORY |
| 10 | 1 | 351 | SATISFACTORY |  | 10 | 0 | 562 | SATISFACTORY |
| 10 | 1 | 32 | SATISFACTORY |  | 10 | 1 | 1000 | SATISFACTORY |
| 10 | 0 | 316 | SATISFACTORY |  | 10 | 0 | 562 | SATISFACTORY |
| 10 | 0 | 316 | SATISFACTORY |  | 10 | 0 | 2512 | SATISFACTORY |
| 10 | 0 | 316 | SATISFACTORY |  | 10 | 1 | 228 | SATISFACTORY |
| 10 | 0 | 32 | SATISFACTORY |  | 10 | 0 | 3162 | SATISFACTORY |
| 10 | 0 | 562 | SATISFACTORY |  | 10 | 0 | 147 | SATISFACTORY |
| 10 | 1 | 100 | SATISFACTORY |  | 10 | 0 | 2064 | SATISFACTORY |
| 10 | 1 | 302 | SATISFACTORY |  | 10 | 0 | 631 | SATISFACTORY |
| 10 | 0 | 1468 | SATISFACTORY |  | 10 | 1 | 1000 | SATISFACTORY |
| 10 | 1 | 351 | SATISFACTORY |  | 10 | 0 | 5623 | SATISFACTORY |
| 10 | 0 | 316 | SATISFACTORY |  | 10 | 1 | 1000 | SATISFACTORY |
| 10 | 0 | 2371 | SATISFACTORY |  | 10 | 0 | 3511 | SATISFACTORY |
| 10 | 1 | 316 | SATISFACTORY |  | 10 | 0 | 208 | SATISFACTORY |
| 10 | 1 | 422 | SATISFACTORY |  | 10 | 0 | 178 | SATISFACTORY |
| 10 | 0 | 2336 | SATISFACTORY |  | 10 | 0 | 492 | SATISFACTORY |
| 10 | 0 | 3162 | SATISFACTORY |  | 10 | 0 | 2198 | SATISFACTORY |
| 10 | 1 | 32 | SATISFACTORY |  | 10 | 0 | 562 | SATISFACTORY |
| 10 | 0 | 562 | SATISFACTORY |  | 10 | 2 | 2371 | SATISFACTORY |
| 10 | 2 | 15 | SATISFACTORY |  | 10 | 0 | 1778 | SATISFACTORY |
| 10 | 1 | 422 | SATISFACTORY |  | 10 | 0 | 215 | SATISFACTORY |
| 10 | 0 | 428 | SATISFACTORY |  | 10 | 0 | 351 | SATISFACTORY |
| 10 | 0 | 1778 | SATISFACTORY |  | 10 | 0 | 3162 | SATISFACTORY |
| 10 | 0 | 3162 | SATISFACTORY |  | 10 | 0 | 316 | SATISFACTORY |
| 10 | 0 | 1778 | SATISFACTORY |  | 10 | 0 | 5623 | SATISFACTORY |
| 10 | 1 | 100 | SATISFACTORY |  | 10 | 0 | 1000 | SATISFACTORY |
| 10 | 0 | 2371 | SATISFACTORY |  | 10 | 0 | 316 | SATISFACTORY |
| 10 | 1 | 481 | SATISFACTORY |  | 10 | 0 | 1778 | SATISFACTORY |
| 10 | 0 | 3162 | SATISFACTORY |  | 10 | 1 | 59 | SATISFACTORY |
| 10 | 1 | 422 | SATISFACTORY |  | 10 | 0 | 1778 | SATISFACTORY |
| 10 | 0 | 10 | SATISFACTORY |  | 10 | 0 | 3162 | SATISFACTORY |
| 10 | 0 | 5623 | SATISFACTORY |  | 10 | 0 | 178 | SATISFACTORY |
| 10 | 0 | 1000 | SATISFACTORY |  | 10 | 0 | 562 | SATISFACTORY |
| 10 | 0 | 1778 | SATISFACTORY |  | 10 | 0 | 3162 | SATISFACTORY |
| 10 | 0 | 18 | SATISFACTORY |  | 10 | 0 | 3162 | SATISFACTORY |
| 10 | 0 | 316 | SATISFACTORY |  | 10 | 0 | 316 | SATISFACTORY |
| 10 | 0 | 2154 | SATISFACTORY |  | 10 | 2 | 228 | SATISFACTORY |
| 10 | 0 | 215 | SATISFACTORY |  | 10 | 1 | 1000 | SATISFACTORY |
| 10 | 2 | 681 | SATISFACTORY |  | 10 | 0 | 1778 | SATISFACTORY |
| 10 | 0 | 316 | SATISFACTORY |  | 10 | 0 | 562 | SATISFACTORY |
| 10 | 0 | 1000 | SATISFACTORY |  | 10 | 1 | 427 | SATISFACTORY |
| 10 | 0 | 316 | SATISFACTORY |  | 10 | 0 | 1778 | SATISFACTORY |
| 10 | 0 | 1995 | SATISFACTORY |  | 10 | 2 | 200 | SATISFACTORY |
| 10 | 0 | 3162 | SATISFACTORY |  | 10 | 1 | 215 | SATISFACTORY |
| 10 | 0 | 562 | SATISFACTORY |  | 10 | 0 | 351 | SATISFACTORY |
| 10 | 0 | 178 | SATISFACTORY |  | 10 | 0 | 1000 | SATISFACTORY |
| 10 | 2 | 1000 | SATISFACTORY |  | 10 | 0 | 3162 | SATISFACTORY |
| 10 | 0 | 1778 | SATISFACTORY |  | 10 | 0 | 1479 | SATISFACTORY |
| 10 | 0 | 316 | SATISFACTORY |  | 10 | 0 | 562 | SATISFACTORY |
| 10 | 0 | 5495 | SATISFACTORY |  | 10 | 0 | 1778 | SATISFACTORY |
| 10 | 0 | 3162 | SATISFACTORY |  | 10 | 0 | 1000 | SATISFACTORY |
| 10 | 1 | 351 | SATISFACTORY |  | 10 | 0 | 2371 | SATISFACTORY |
| 10 | 0 | 1000 | SATISFACTORY |  | 10 | 1 | 215 | SATISFACTORY |
| 10 | 0 | 3162 | SATISFACTORY |  | 10 | 0 | 316 | SATISFACTORY |
| 10 | 2 | 1000 | SATISFACTORY |  | 10 | 0 | 1778 | SATISFACTORY |
| 10 | 0 | 46 | SATISFACTORY |  | 10 | 0 | 3162 | SATISFACTORY |
| 10 | 1 | 1000 | SATISFACTORY |  | 10 | 0 | 562 | SATISFACTORY |
| 10 | 0 | 3162 | SATISFACTORY |  | 10 | 0 | 562 | SATISFACTORY |
| 10 | 1 | 32 | SATISFACTORY |  | 10 | 0 | 215 | SATISFACTORY |
| 10 | 1 | 2154 | SATISFACTORY |  | 10 | 0 | 373 | SATISFACTORY |
| 10 | 1 | 32 | SATISFACTORY |  | 10 | 1 | 228 | SATISFACTORY |
| 10 | 1 | 250 | SATISFACTORY |  | 10 | 1 | 1468 | SATISFACTORY |
| 10 | 0 | 316 | SATISFACTORY |  | 10 | 0 | 316 | SATISFACTORY |
| 10 | 0 | 562 | SATISFACTORY |  | 10 | 0 | 1000 | SATISFACTORY |
| 10 | 0 | 304 | SATISFACTORY |  | 10 | 0 | 170 | SATISFACTORY |
| 10 | 1 | 3162 | SATISFACTORY |  | 10 | 0 | 464 | SATISFACTORY |
| 10 | 0 | 316 | SATISFACTORY |  | 10 | 0 | 3162 | SATISFACTORY |
| 10 | 0 | 10000 | SATISFACTORY |  | 10 | 0 | 794 | SATISFACTORY |
| 10 | 1 | 1000 | SATISFACTORY |  | 10 | 0 | 1995 | SATISFACTORY |
| 10 | 0 | 215 | SATISFACTORY |  | 10 | 0 | 562 | SATISFACTORY |
| 10 | 0 | 2154 | SATISFACTORY |  | 10 | 1 | 215 | SATISFACTORY |
| 10 | 0 | 178 | SATISFACTORY |  | 10 | 0 | 316 | SATISFACTORY |
| 10 | 0 | 351 | SATISFACTORY |  | 10 | 0 | 3162 | SATISFACTORY |
| 10 | 0 | 68 | SATISFACTORY |  | 10 | 1 | 316 | SATISFACTORY |
| 10 | 0 | 32 | SATISFACTORY |  | 10 | 0 | 316 | SATISFACTORY |
| 10 | 0 | 40 | SATISFACTORY |  | 10 | 0 | 3162 | SATISFACTORY |
| 10 | 0 | 3162 | SATISFACTORY |  | 10 | 1 | 228 | SATISFACTORY |
| 10 | 0 | 492 | SATISFACTORY |  | 10 | 1 | 955 | SATISFACTORY |
| 10 | 0 | 1334 | SATISFACTORY |  | 10 | 0 | 316 | SATISFACTORY |
| 10 | 0 | 178 | SATISFACTORY |  | 10 | 1 | 178 | SATISFACTORY |
| 10 | 0 | 3162 | SATISFACTORY |  | 10 | 0 | 215 | SATISFACTORY |
| 10 | 0 | 1000 | SATISFACTORY |  | 10 | 1 | 178 | SATISFACTORY |
| 10 | 0 | 178 | SATISFACTORY |  | 10 | 0 | 351 | SATISFACTORY |
| 10 | 2 | 1000 | SATISFACTORY |  | 10 | 1 | 228 | SATISFACTORY |
| 10 | 1 | 1778 | SATISFACTORY |  | 10 | 0 | 562 | SATISFACTORY |
| 10 | 0 | 2089 | SATISFACTORY |  | 10 | 0 | 481 | SATISFACTORY |
| 10 | 0 | 562 | SATISFACTORY |  | 10 | 0 | 147 | SATISFACTORY |
| 10 | 0 | 316 | SATISFACTORY |  | 10 | 0 | 251 | SATISFACTORY |
| 10 | 1 | 151 | SATISFACTORY |  | 10 | 0 | 3162 | SATISFACTORY |
| 10 | 1 | 351 | SATISFACTORY |  | 10 | 0 | 3162 | SATISFACTORY |
| 10 | 0 | 4786 | SATISFACTORY |  | 10 | 0 | 316 | SATISFACTORY |
| 10 | 1 | 3981 | SATISFACTORY |  | 10 | 0 | 3162 | SATISFACTORY |
| 10 | 0 | 3162 | SATISFACTORY |  | 10 | 0 | 1000 | SATISFACTORY |
| 10 | 0 | 1479 | SATISFACTORY |  | 10 | 0 | 562 | SATISFACTORY |
| 10 | 0 | 479 | SATISFACTORY |  | 10 | 0 | 351 | SATISFACTORY |
| 10 | 1 | 491 | SATISFACTORY |  | 10 | 0 | 174 | SATISFACTORY |
| 10 | 0 | 316 | SATISFACTORY |  | 10 | 0 | 351 | SATISFACTORY |
| 10 | 1 | 2154 | SATISFACTORY |  | 10 | 0 | 178 | SATISFACTORY |
| 10 | 0 | 316 | SATISFACTORY |  | 10 | 0 | 3162 | SATISFACTORY |
| 10 | 0 | 179 | SATISFACTORY |  | 10 | 1 | 1000 | SATISFACTORY |
| 10 | 0 | 562 | SATISFACTORY |  | 10 | 1 | 1000 | SATISFACTORY |
| 10 | 0 | 6471 | SATISFACTORY |  | 10 | 1 | 501 | SATISFACTORY |
| 10 | 0 | 178 | SATISFACTORY |  | 10 | 0 | 316 | SATISFACTORY |
| 10 | 0 | 3162 | SATISFACTORY |  | 10 | 0 | 3511 | SATISFACTORY |
| 10 | 0 | 32 | SATISFACTORY |  | 10 | 0 | 562 | SATISFACTORY |
| 10 | 0 | 178 | SATISFACTORY |  | 10 | 1 | 1334 | SATISFACTORY |
| 10 | 0 | 1000 | SATISFACTORY |  | 10 | 0 | 1000 | SATISFACTORY |
| 10 | 1 | 302 | SATISFACTORY |  | 10 | 0 | 316 | SATISFACTORY |
| 10 | 0 | 562 | SATISFACTORY |  | 10 | 0 | 603 | SATISFACTORY |
| 10 | 1 | 2154 | SATISFACTORY |  | 10 | 1 | 228 | SATISFACTORY |
| 10 | 0 | 316 | SATISFACTORY |  | 10 | 0 | 316 | SATISFACTORY |
| 10 | 1 | 178 | SATISFACTORY |  | 10 | 1 | 165 | SATISFACTORY |
| 10 | 0 | 316 | SATISFACTORY |  | 10 | 1 | 228 | SATISFACTORY |
| 10 | 0 | 316 | SATISFACTORY |  | 10 | 0 | 562 | SATISFACTORY |
| 10 | 1 | 178 | SATISFACTORY |  | 10 | 0 | 1778 | SATISFACTORY |
| 10 | 0 | 562 | SATISFACTORY |  | 10 | 0 | 3162 | SATISFACTORY |
| 10 | 0 | 316 | SATISFACTORY |  | 10 | 0 | 562 | SATISFACTORY |
| 10 | 1 | 422 | SATISFACTORY |  | 10 | 0 | 3162 | SATISFACTORY |
| 10 | 0 | 316 | SATISFACTORY |  | 10 | 1 | 178 | SATISFACTORY |
| 10 | 1 | 100 | SATISFACTORY |  | 10 | 0 | 422 | SATISFACTORY |
| 10 | 0 | 681 | SATISFACTORY |  | 10 | 0 | 10000 | SATISFACTORY |
| 10 | 0 | 1778 | SATISFACTORY |  | 10 | 0 | 178 | SATISFACTORY |
| 10 | 0 | 10000 | SATISFACTORY |  | 10 | 0 | 1000 | SATISFACTORY |
| 10 | 0 | 3875 | SATISFACTORY |  | 10 | 0 | 351 | SATISFACTORY |
| 10 | 1 | 422 | SATISFACTORY |  | 10 | 0 | 1778 | SATISFACTORY |
| 10 | 0 | 5623 | SATISFACTORY |  | 10 | 1 | 178 | SATISFACTORY |
| 10 | 1 | 100 | SATISFACTORY |  | 10 | 0 | 316 | SATISFACTORY |
| 10 | 1 | 100 | SATISFACTORY |  | 10 | 1 | 316 | SATISFACTORY |
| 10 | 0 | 562 | SATISFACTORY |  | 10 | 0 | 2371 | SATISFACTORY |
| 10 | 0 | 562 | SATISFACTORY |  | 10 | 0 | 215 | SATISFACTORY |
| 10 | 1 | 100 | SATISFACTORY |  | 10 | 0 | 316 | SATISFACTORY |
| 10 | 1 | 1000 | SATISFACTORY |  | 10 | 2 | 3162 | SATISFACTORY |
| 10 | 1 | 32 | SATISFACTORY |  | 10 | 0 | 3162 | SATISFACTORY |
| 10 | 1 | 55 | SATISFACTORY |  | 10 | 0 | 3162 | SATISFACTORY |
| 10 | 0 | 562 | SATISFACTORY |  | 10 | 0 | 562 | SATISFACTORY |
| 10 | 1 | 215 | SATISFACTORY |  | 10 | 1 | 422 | SATISFACTORY |
| 10 | 1 | 215 | SATISFACTORY |  | 10 | 1 | 2154 | SATISFACTORY |
| 10 | 0 | 316 | SATISFACTORY |  | 10 | 0 | 562 | SATISFACTORY |
|  |  |  |  |  | 10 | 1 | 234 | SATISFACTORY |
|  |  |  |  |  | 10 | 0 | 955 | SATISFACTORY |
|  |  |  |  |  | 10 | 1 | 501 | SATISFACTORY |
|  |  |  |  |  | 10 | 2 | 228 | SATISFACTORY |
|  |  |  |  |  | 10 | 1 | 178 | SATISFACTORY |
